# Effect of Sodium Dodecyl Sulfate on Stability of MXene Aqueous Dispersion

**DOI:** 10.1002/advs.202300273

**Published:** 2023-06-22

**Authors:** Baomin Fan, Xiaoqi Zhao, Peng Zhang, Yi Wei, Ning Qiao, Biao Yang, Razium A. Soomro, Ran Zhang, Bin Xu

**Affiliations:** ^1^ College of Chemical and Materials Engineering Beijing Technology and Business University Beijing 100048 China; ^2^ State Key Laboratory of Organic–Inorganic Composites Beijing Key Laboratory of Electrochemical Process and Technology for Materials Beijing University of Chemical Technology Beijing 100029 China

**Keywords:** 2D MXenes, antioxidants, oxidation stabilities, sodium dodecyl sulfate (SDS), theoretical modelings

## Abstract

MXenes suffer from severe oxidation and progressive degradation in aqueous media due to its poor chemical stability. Herein, sodium dodecyl sulfate (SDS) is employed as an efficient protectant for long‐term storage of Ti_3_C_2_T*_x_*‐MXene aqueous dispersion. Experimental data support SDS's capability to protect oxidation‐prone sites on Ti_3_C_2_T*_x_* nanosheets, providing extended colloidal stability of up to 213 days. Concentration‐dependent anti‐oxidation effect articulates that 1.5 mg mL^−1^ is deemed as an ideal SDS dose for Ti_3_C_2_T*_x_* to achieve optimal oxidation‐resistance in aqueous solution. Additionally, a chroma strategy is developed to instantly and precisely measure the oxidation degree of Ti_3_C_2_T*_x_*. Adsorption‐driven anti‐oxidation efficacy of SDS is further confirmed by optimized conformations with interaction energies of SDS on termination‐free and surface‐defective Ti_3_C_2_T*_x_* through multiscale simulations. This proposed route is a step forward in broadening the horizons of experimental and theoretical investigations of MXenes with promising implications for long‐term storage and reliable applications.

## Graphical abstract

Sodium dodecyl sulfate (SDS) recognizes and adsorbs on the oxidation‐sensitive defects and edges of Ti_3_C_2_T*_x_* nanosheets in the aqueous dispersion, and thus effectively inhibits its oxidation advancement based on the matched polarity and charge compatibility. Critical micelle concentration (CMC) is a threshold of SDS for the oxidation‐resistance of Ti_3_C_2_T*_x_* nanosheets in the aqueous solution.

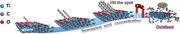

## 1 Introduction

Transition metal carbides and/or nitrides, also known as MXenes, are a class of 2D materials that are rapidly expanding due to their attractive properties, such as metallic electrical/thermal conductivity, diversified surface chemistry, and favorable hydrophilicity. Besides energy storage, MXenes have drawn increasing attention in various domains, including biotherapy, sensors, catalysis, and electromagnetic interference shielding.^[^ ^1^ ^]^ In their general formula M*_n+_*_1_X*_n_* T*_x_*, M denotes early transition metals (e.g., Ti, Nb, Mo, and V), X denotes carbon and/or nitrogen, T represents surface terminals (—O, —F, —OH, etc.), and *n* is 1–4.^[^ ^2^ ^]^ Diverse strategies have been developed to obtain MXenes via selective etching of MAX phase precursors (A: an element mainly from group 13 or 14) employing suitable etchant formulae. Combining wet‐etching and delamination procedures for preparing MXenes in aqueous colloids is apparently competitive depending on the facile operation, cost‐effective, good quality in single or few‐layers morphology, and direct achievement in high‐concentration dispersion,^[^ ^3^ ^]^ which is feasible for their integration into other systems and exploration of broad applications. Despite the excellent physicochemical properties, MXenes are prone to oxidation in aqueous media, causing impaired bonds and progressive collapse of 2D architectures. For the most studied titanium carbide (i.e., Ti_3_C_2_T*_x_*), hydrofluoric acid (HF) is the earliest etchant reported by Gogotsi and Barsoum to remove the aluminum layer from Ti_3_AlC_2_, yielding the accordion‐like MXene powder.^[^ ^4^ ^]^ However, the corrosive HF results in nanoscale defects, such as lattice vacancies on the basal plane or edges of exfoliated Ti_3_C_2_T*_x_*. Even etching with relatively mild alternatives, such as in situ formed HF from HCl/LiF couple, bifluoride salts, and alkali‐system, MXene's surface still has sufficient imperfections to permit chronic oxidation and progressive degradation.

Hitherto, multiple concurrent reactions have been proposed to understand the initiation of MXene oxidation leading to the sophisticated mechanism that requires in‐depth elucidation. Among the widely‐accepted concepts, edges of MXene nanosheets are more susceptible to oxidation than the basal plane due to totally exposed defects,^[^ ^5^ ^]^ and thus edge‐defects act as vulnerable centers for the further development of oxidation in aqueous media. Natu et al. proved that the positively charged edges and planar defects triggered Ti_3_C_2_T*_x_* oxidation in aqueous media.^[^ ^6^ ^]^ Consequently, an internal electric field is formed between positive Ti vacancies and nearby C^4−^ ions, providing transit channels for charge carriers.^[^ ^7^ ^]^ Moreover, the oxidation initiated at defects whereby the unimpeded electron transportation between the Ti‐rich region and C layer is rapidly aggravated, resulting in the growth of lattice voids and TiO_2_ grains along with the disintegration of the Ti—C backbone.^[^ ^8^ ^]^ In aqueous media, the dissolved oxygen and protons from water would attack the oxidation‐susceptible sites, favoring the acid‐catalyzed hydrolysis of Ti_3_C_2_T*_x_*.^[^ ^9^ ^]^ This rate‐determining step, influenced by the concurrent oxidation and hydrolysis reactions, is a bottleneck problem that highly limits the large‐scale synthesis and practical applications of MXenes. Therefore, it is urgent to overcome the degradation issue of MXenes to guarantee their good electric/electrochemical performances.

Generally, high‐stoichiometric MXenes species and concentrated colloids with larger sheet dimensions relatively own an ambient stability for several months.^[^ ^10^ ^]^ Nonetheless, concentrated MXene dispersions cannot fundamentally cure the destined premature oxidation. Thereupon, persistent efforts have been made to improve the oxidation resistance of acquired MXene colloids. Presently, three common post‐synthesis processes are in service to prolong the shelf‐life of MXene dispersions: storage condition regulation, surface covalent modification, and using antioxidants.^[^ ^8^, ^10^ ^]^ Despite its effectiveness, regulating the preservation condition, such as cold‐storing MXene aqueous dispersions at ultra‐low temperatures (e.g., −80°C) within inert gas,^[^ ^6^, ^11^ ^]^ is not an energy‐saving route for large‐scale operations. Similarly, preserving MXenes in organic solvents (e.g., dimethyl formamide) also mitigates their oxidation; however, the aqueous re‐dispersibility and surface reactivity are compromised in practice.^[^ ^12^ ^]^ In addition, using strong brine as the storage medium has been reported to prolong the shelf‐life of Ti_3_C_2_T*_x_* up to 400 days,^[^ ^9a^ ^]^ but the elimination of salt to recover MXenes in their natural form tends to be an energy consuming and tedious procedure for the actual application. Surface covalent stabilization by polyurethane has also been proven effective in retarding the oxidation kinetics of Ti_3_C_2_T*_x_* in water.^[^ ^13^ ^]^ Unfortunately, the covalent modification would inevitably result in surface‐charge conversion, which might interfere with the electrochemical characteristics of MXenes, particularly adverse to their energy storage applications. Likewise, Ti_3_C_2_T*_x_* nanosheets could be modified by silk fibroin to improve their water stability, albeit this still requires sophisticated transformation protocols with compromised applicability.^[^ ^14^ ^]^

Recently, anti‐oxidants, such as L‐ascorbate, citrate, and polyanionic salts have been utilized to mitigate MXene oxidation in water.^[^ ^15^ ^]^ These added small molecules have gained tremendous recognition for their availability and high efficiency. Although the detailed anti‐oxidative mechanism is still ambiguous, it is proven that antioxidant anions could cap the positively charged edges/defects, thus alleviating the oxidation of MXenes. For instance, the shelf‐life for Ti_3_C_2_T*_x_* with these additives has attained 21 days keeping a certain dispersity. However, the deep science of these edge‐capping agents for stabilizing MXene is still in its infancy, and the oxidation mechanism in MXenes is presently under debate. According to the latest literature, the oxidation and hydrolysis of Ti_3_C_2_T*_x_* in water define O_2_ and H_2_O as the respective culprit to initiate the degradation.^[^ ^10c^ ^]^ Thus, it is still a critical challenge to identify an efficient anti‐oxidation agent for the long‐term storage of MXenes and validate the protection mechanism.

**Figure 1:**
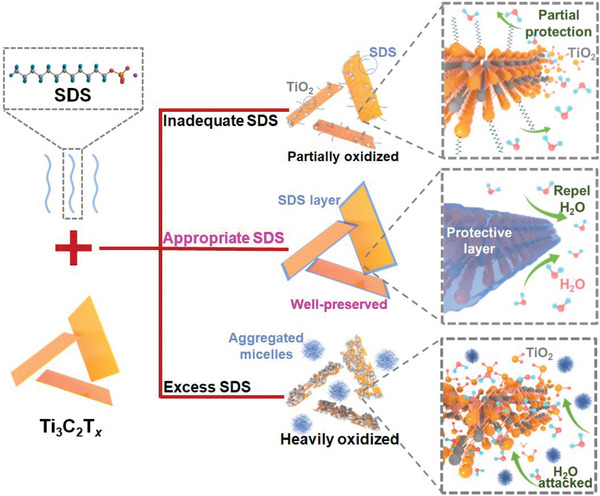
Schematic illustration of SDS‐induced anti‐oxidation capability in Ti_3_C_2_T*_x_* colloidal dispersion at varied SDS concentrations.

Conceivably, if a compound not only shielded the defects, but also minimized the solvent‐MXene interfacial area, such an anti‐oxidant imparting anti‐oxidation. It is therefore, we believe anionic surfactants may be potential candidates for inhibiting MXenes oxidation in aqueous media: the anionic terminal may interact with positively charged MXene edges and defects; while the alkyl tails may crosswise assemble over MXenes and function as a protective layer. Herein, we report a facile route to stabilize Ti_3_C_2_T*_x_* aqueous colloid using sodium dodecyl sulfate (SDS) as an efficient anti‐oxidant. At an optimal dosage of SDS, the anions (SDS^−^) can effectively adhere to the oxidation‐susceptible sites on Ti_3_C_2_T*_x_* nanosheets through non‐ligand electrons, enabling well‐preservation of MXene nanosheets and extending its colloidal stability. It is found the stability of MXene depends strongly on the added concentration of SDS as illustrated in **Figure** **1**. Insufficient SDS hardly establishes the required oxidation resistance of Ti_3_C_2_T*_x_* dispersion for the incomplete coverage of SDS^−^ anions on the vulnerable defects; conversely, excess concentration of SDS is detrimental to MXene stability due to aggregation of the surfactant. Monte Carlo (MC), molecular dynamics (MD) simulations and density functional theory (DFT) calculations were employed to probe the possible interaction between SDS and Ti_3_C_2_T*_x_*. Particular attention was poured on electron transport between SDS and MXene defects to clarify the adsorption behavior. In contrast to other ionic analogues, the optimized SDS feeding ratio to stabilize Ti_3_C_2_T*_x_* is relatively low. At an optimal 1.5 mg mL^−1^ SDS, the structural integrity of Ti_3_C_2_T*_x_* nanosheets could be well‐preserved for a shelf‐life of 213 days (experimental limit). The proposed approach is a step forward in providing a systematic foundation to bridge the gap between theoretical and experimental evidence for the science of ionic stabilization toward MXenes, with promising implications for their long‐term colloidal storage without oxidation and the physicochemical potential for broad‐spectrum applications.

## 2 Results and Discussion

**Figure 2:**
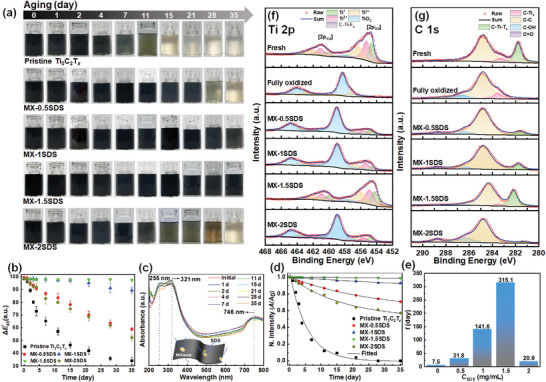
Color degradation of Ti_3_C_2_T*_x_* suspensions without and with different concentrations of a) SDS attached by the deviations of b) chroma values ($\Delta E_{\mathrm{ab}}^{*}$) over time; UV–vis spectra for the suspensions with 1.5 mg mL^−1^ SDS at c) the allocated time intervals along with the fitting curves of d) colloidal stability and the related time constants (*τ*) for unprotected and protected Ti_3_C_2_T*_x_* suspensions; deconvoluted XPS spectra of f) Ti 2*p* and g) C 1*s* for Ti_3_C_2_T*_x_* vacuum‐filtered films obtained from freshly etched, unprotected and protected Ti_3_C_2_T*_x_* solutions with different SDS concentrations after 35 days of aging.

To investigate the potential of SDS as an efficient antioxidant for MXene and to assess its optimal dose, a concentration gradient of 0.5, 1.0, 1.5, and 2.0 mg mL^−1^ was set for stabilizing Ti_3_C_2_T*_x_* colloids (0.05 mg mL^−1^) with corresponding MXene dispersions denoted as MX‐0.5SDS, MX‐1SDS, MX‐1.5SDS, and MX‐2SDS, respectively. **Figure** **2**a shows the color deterioration of Ti_3_C_2_T*_x_* dispersions containing different SDS doses in reference to the fresh control during 35 days of naturally‐aerated aging at 298±1.5 K. As seen, the signature dark color of pristine Ti_3_C_2_T*_x_* slowly decays to blackish green and subsequently translucent after 7 and 15 days, respectively. Color fading is a visible evidence of MXene's ongoing oxidation and/or hydrolysis in aqueous environments. By comparison, the dispersions containing SDS in a concentration range from 0.5 to 1.5 mg mL^−1^ can retain the black feature for a longer duration than the fresh control, suggesting a retarded degradation kinetics.^[^ ^16^ ^]^ Remarkably, MX‐1.5SDS exhibits the negligible color change even after 35 days; however, a higher SDS dose (2.0 mg mL^−1^) ruins the established stability and results in a greenish color after 11 days, which finally becomes hazy white after 35 days.

The color variation is frequently regarded as an unmistakable indication to assess the degradation degree of MXenes. Thus, we propose, for the first time, a chroma strategy to quantitatively evaluate the stability of Ti_3_C_2_T*_x_* dispersions. Total chroma ($\Delta E_{\mathrm{ab}}^{*}$) was recorded with aging time for different dispersions, allowing for a precise measurement of color change and thus the degradation degree of Ti_3_C_2_T*_x_*. In Figure 2b, $\Delta E_{\mathrm{ab}}^{*}$ for the pristine MXene dispersion declines rapidly with time, indicating the drastic color change caused by Ti_3_C_2_T*_x_* degradation. In contrast, the attenuation of $\Delta E_{\mathrm{ab}}^{*}$ is increasingly delayed as the SDS content rises from 0.5 to 1.5 mg mL^−1^. Particularly for MX‐1.5SDS, there is a negligible decay in $\Delta E_{\mathrm{ab}}^{*}$ even after 35 days, confirming the efficient oxidation resistance of MXene rendered by SDS. However, when SDS concentration rises to 2.0 mg mL^−1^, $\Delta E_{\mathrm{ab}}^{*}$ drops low, revealing the attenuated oxidation stability. To further verify the anti‐oxidative efficacy, the evolution of UV–vis spectra with aging time was monitored for Ti_3_C_2_T*_x_* dispersions with different SDS contents. Generally, Ti_3_C_2_T*_x_* exhibits distinctive bands at 746 and 321 nm with a thermodynamically preferred oxidation band at 255 nm assigned to *n*→*σ* ^*^ transition of Ti—O,^[^ ^16^ ^]^ and the attenuation of these bands implies MXene aggregation and degradation. For pristine Ti_3_C_2_T*_x_* (Figure S1a, Supporting Information), the suppressed spectrum is evident with the disappearance of bands at 746 and 321 nm and a distinguishable band at 255 nm, suggesting the formation, aggregation and sedimentation of TiO_2_.^[^ ^13^, ^16^, ^17^ ^]^ Progressively improved anti‐oxidation effect of SDS is revealed in Figure S1b,c (Supporting Information) for the respective MX‐0.5SDS and MX‐1.0SDS. Notably, UV–vis spectra measured throughout the aging period for MX‐1.5SDS (Figure 2c) display a comparatively slow absorbance decline with minute band change at 255 nm. As anticipated, when SDS content exceeds 2 mg mL^−1^, both absorbances at 321 and 746 nm decrease due to inadequate protection against oxidation (Figure S1d, Supporting Information).

The stability of Ti_3_C_2_T*_x_* dispersion was quantitatively assessed by the first‐order kinetics from their typical UV–vis absorbances at 746 nm using the following equation:^[^ ^5^, ^16^ ^]^

(1) $$A=A_{\mathrm{unoxd}}+A_{\mathrm{re}}e^{\left(-t/\tau \right)}=A_{\mathrm{unoxd}}+A_{\mathrm{re}}e^{\left(-kt\right)}$$

where *A*, *A*_unoxd_, and *A*_re_ denote the absorbances of Ti_3_C_2_T*_x_* dispersions after a pre‐set aging interval, unreacted and reacted analogues, respectively; *t* denotes the oxidation time (h); *τ* signifies the decay constant denoting the time required for MXene decay to 36.8% (1/*e*), while *k* represents the reaction rate constant. Kinetic fittings are summarized in Figure 2d, and the estimated *τ* values are plotted versus SDS contents in Figure 2e. Clearly, *τ* for unprotected Ti_3_C_2_T*_x_* is only 7.5 days, similar to those previously reported.^[^ ^16^, ^18^ ^]^ For SDS‐stabilized dispersions, elevated *τ* values of 31.8, 141.6, and 315.1 days are achieved for MX‐0.5SDS, MX‐1SDS, and MX‐1.5SDS, respectively, which plummets to 20.9 days for MX‐2SDS. Notably, the degradation kinetics in Figure 2d follows the typical pattern of $\Delta E_{\mathrm{ab}}^{*}$ variation (Figure 2b), and thus further supports the reliability of chroma strategy as a promising candidate to evaluate the colloidal stability of MXenes.

The oxidation of MXenes may experience an acid‐catalyzed route that initiates from the positively charged defects.^[^ ^5^ ^]^ It is possible for SDS^−^ in water to migrate to, and adsorb on the edge and/or lateral defects of Ti_3_C_2_T*_x_* nanosheets through Coulomb attraction (inset of Figure 2c), shielding of oxidation‐susceptible sites. Besides, the stacked aliphatic tails among SDS^−^ chains prefer sealing the edges, thus protecting the lateral flaws of Ti_3_C_2_T*_x_*. Thereby, within a certain range, the anti‐oxidative effect is progressively reinforced as the SDS dosage increases. With the appropriate concentration of SDS (1.5 mg mL^−1^), the degradation of Ti_3_C_2_T*_x_* is significantly suppressed, and the maintained charge distribution of the uncovered Ti_3_C_2_T*_x_* portion further preserves the colloidal stability, assuring little sedimentation even after 213 days under lab ambient. This is further confirmed by the Tyndall effect (Figure S2, Supporting Information), UV–vis spectrum retention of MX‐1.5SDS (Figure S3a, Supporting Information) and the intact 2D morphology (Figure S3b, Supporting Information) with the well‐reserved crystal edge (Figure S3c, Supporting Information). It is well‐established that oxidation of MXene proceeds more easily at lower aqueous concentrations. As shown in Table S1 (Supporting Information), MXene aqueous solution with an ultralow concentration of 0.05 mg mL^−1^ may be stored for up to 213 days with a modest SDS dose of 1.5 mg mL^−1^, much superior to the anti‐oxidative effect of some previously reported antioxidants, such as polyphosphate (36.8 and 15 mg mL^−1^ of Ti_3_C_2_T*_x_*, 21 days),^[^ ^15a^ ^]^ sodium L‐ascorbate (2.0 and 0.2 mg mL^−1^ of Ti_3_C_2_T*_x_*, 80 days),^[^ ^15b^ ^]^ and sodium citrate (1.5 and 0.3 mg mL^−1^ of Ti_3_C_2_T*_x_*, 150 days).^[^ ^15c^ ^]^ On the other hand, insufficient oxidation‐resistance of MX‐2SDS could be ascribed to the critical micelle concentration (CMC) of SDS. In this regard, CMC value for SDS in water was determined to be 1.91 mg mL^−1^ through conductivity monitoring (Figure S4, Supporting Information); thereupon, at 2.0 mg mL^−1^ (over CMC), poor dispersity of SDS in MXene dispersion impedes the effective defect‐capping due to the dominant repulsion between negatively charged Ti_3_C_2_T*_x_* plane and surfactant micelles.

To further quantify the anti‐oxidative effect of SDS for Ti_3_C_2_T*_x_*, XPS explorations were performed on vacuum‐filtered MXene films from the dispersions with varying SDS contents after 35 days of aging. Figure 2f shows Ti 2*p* spectra for SDS‐associated films in reference to pristine and completely oxidized specimens. For fresh Ti_3_C_2_T*_x_*, typical Ti 2*p*_1/2_ and Ti 2*p*_3/2_ (in brackets) doublets were determined at 454.68 (460.66), 455.28 (460.93), 456.38 (462.11), 458.08, and 458.87 eV for Ti^+^ (Ti—C), Ti^2+^, Ti^3+^ (Ti*_x_* O*_y_*), TiO_2_, and C—Ti—F*_x_*, respectively.^[^ ^15^, ^19^ ^]^ A significant TiO_2_ contribution is evident for the unprotected Ti_3_C_2_T*_x_* sample, where ≈93.6% of total Ti can be assigned to TiO_2_ as assessed in Table S2 (Supporting Information). Binding energies related to Ti_3_C_2_T*_x_* backbone, i.e., Ti^+^ (Ti—C), Ti^2+^, and Ti^3+^ in Ti 2*p*_3/2_ moiety, are stable with the optimum anti‐oxidant dose. The greatest Ti—C and the least TiO_2_ (4.9%) contents are identified for MX‐1.5SDS, which also justifies 1.5 mg mL^−1^ SDS to retain nearly the same typical photoelectron contributions as fresh Ti_3_C_2_T*_x_*. With respect to C 1*s* spectra in Figure 2g, typical peaks at 281.76, 283.27, and 284.79 eV for fresh MXene could be indexed to C—Ti—T*_x_*, C—Ti—O, and C—C, respectively.^[^ ^11^, ^20^ ^]^ Without SDS protection, C—Ti—T*_x_* disappears after 35 days of storage, revealing the complete oxidation of MXenes with an additional peak at 288.82 eV for C=O.^[^ ^21^ ^]^ After adding SDS, the associated C 1*s* spectra are devoid of C=O species, suggesting enhanced colloidal stability and structural integrity. Figure S5 (Supporting Information) shows the O1*s* spectrum of fresh Ti_3_C_2_T*_x_* consisting of primary binding energies at 531.01 and 532.20 eV attributed to C—Ti—O*_x_* and C—Ti—(OH)*_x_*, respectively.^[^ ^22^ ^]^ An apparent TiO_2_ response is recognized for the fully oxidized specimen that confirms the transformation of the Ti—C backbone to TiO_2_ according to the following reaction (taking Ti_3_C_2_O_2_ as an example):^[^ ^10c^ ^]^

(2) $${\mathrm{Ti}}_{3}C_{2}O_{2}+4H_{2}O\to 3{\mathrm{TiO}}_{2}+2C+4H_{2}$$

Moreover, SEM images of vacuum‐filtered films from the aged Ti_3_C_2_T*_x_* dispersions were captured to examine the surface and cross‐sectional morphologies in comparison to their pristine control. Fresh Ti_3_C_2_T*_x_* film has a flexible and crumpled surface (Figure S6a, Supporting Information), which converts into an uneven stack of stiff TiO_2_ after 35 days (Figure S6b, Supporting Information) without protection. An improved surface homogeneity and integrity can be seen in Figure S6c–e (Supporting Information) with rising SDS content (i.e., MX‐0.5SDS, MX‐1SDS, and MX‐1.5SDS). Therein, MX‐1.5SDS achieves a morphology that resembled the fresh control. Cross‐sectional observations of different films also support the superiority of 1.5 mg mL^−1^ SDS in maintaining structural integrity and flexibility for Ti_3_C_2_T*_x_* nanosheets. Unlike the heavy deterioration of pristine film without SDS after aging, MX‐1.5SDS film retains its lamellar property in Figure S7 (Supporting Information), suggesting the inhibited oxidation and preserved structural integrity of Ti_3_C_2_T*_x_* nanosheets by SDS. In contrast, MX‐0.5SDS and MX‐2SDS films exhibit exhausted flexibility with obvious voids and fractures, which may result from the aggregation of rigid TiO_2_ grains.

**Figure 3:**
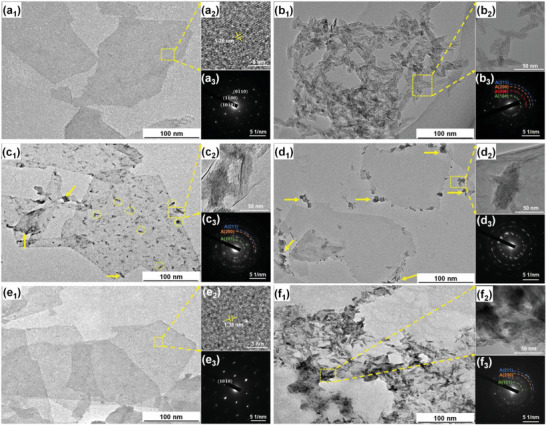
a_1_–f_1_) TEM and a_2_–f_2_) HRTEM, amplification of the yellow‐framed region in (a_1_–f_1_) images of Ti_3_C_2_T*_x_* nanosheet: a) fresh, and the samples after 35 days of b) aging without and with c) 0.5, d) 1.0, e) 1.5, and f) 2.0 mg mL^−1^ SDS along with the SAED patterns in (a_3_–f_3_).

For elucidating the protective efficiency of SDS, TEM analysis was conducted on Ti_3_C_2_T*_x_* dispersions with different SDS contents after 35 days of aging. TEM images of different MXene dispersions are presented in **Figure** **3** along with the corresponding selected area electron diffraction (SEAD). Well‐exfoliated 2D nanosheets can be seen for fresh MXene in Figure 3a1, with an inter‐space of 1.26 nm (Figure 3a2), which refer to the distance between adjacent ordered structures (Figure 3a3). For unprotected dispersions (Figure 3b1), agglomerated particles indicate serious oxidation. In detail, the high‐resolution image in Figure 3b2 confirms the formation of rod‐like anatase TiO_2_ grains with (101), (004), (200), and (211) planes as seen in the SAED pattern (Figure 3b3).^[^ ^23^ ^]^ At a lower SDS dose (MX‐0.5SDS, Figure 3c1), partial oxidation is evident along the flake edge (yellow arrow) and on the basal plane (yellow dotted cycle). While, the oxidation phenomenon is limited to edges when SDS content increases to 1.0 mg mL^−1^ (Figure 3d1), yielding anatase grains along the edge (Figure 3c3,d3). The difference in oxidation resistance of MX‐0.5SDS and MX‐1SDS specimens may be attributed to the favored adsorption of SDS upon oxidation initiation.^[^ ^8^ ^]^ Through electrostatic attraction, SDS‐ preferentially adsorbs on the positively charged defects of the basal plane. As a result, the oxidation‐prone sites on Ti_3_C_2_T*_x_* would be gradually protected as the SDS content augments. Moreover, steric hindrance of alkyl tails on the adsorbed SDS‐ facilitates the shielding of other uncovered susceptible centers. Thus, at an optimal SDS dose (MX‐1.5SDS), the intact lamellar morphology with an equivalent lateral size to that of fresh MXene is observed in Figure 3e1. The corresponding distance between adjacent ordered structures (1.33 nm, Figure 3e3) is close to that of freshly‐exfoliated nanosheet in Figure 3e2. Whereas, excess SDS (2 mg mL^−1^) leads to the structural disintegration (Figure 3f1) accompanied by the formation of amorphous carbon and TiO_2_ (Figure 3f2),^[^ ^18^, ^20^ ^]^ which results from the insufficient protection of SDS micelles. The micro‐/nano‐scale morphological comparison reveals that the presence of excess SDS might be regarded as contamination during practical applications of MXenes.

However, since the evidenced weak contact for SDS on Ti_3_C_2_T*_x_*,^[^ ^24^ ^]^ washing away excess SDS would be straightforward and may have negligible effect on MXenes' physicochemical characteristics. XRD analysis was deployed to verify the compositions of SDS‐capped Ti_3_C_2_T*_x_* films. Figure S8 (Supporting Information) shows XRD patterns of vacuum‐filtered films before and after 35 days of aging with various SDS contents. For fresh MXene, a series of (00*l*) Bragg peaks (Figure S8a, Supporting Information) confirm its well‐aligned 2D architectures.^[^ ^23^ ^]^ A typical sharp peak at 6.13° (2*θ*) is related to the (002) plane of the pristine Ti_3_C_2_T*_x_*, which slightly downshifts to 6.11° in the case of MX‐1.5SDS, implying increased interlayer spacing.^[^ ^15a^ ^]^ This could be credited to the mild intercalation of SDS molecules within the intra‐layer network of MXene nanosheets, in good agreement with TEM image observed for MX‐1.5SDS in Figure 3e2. Unlike MX‐0.5SDS and MX‐1SDS which consist of an anatase phase, MX‐1.5SDS shows little TiO_2_ sign in its pattern. The well‐resolved diffraction feature comparable to that of fresh Ti_3_C_2_T*_x_* consolidates 1.5 mg mL^−1^ SDS as an appropriate concentration for achieving effective anti‐oxidation.

**Figure 4:**
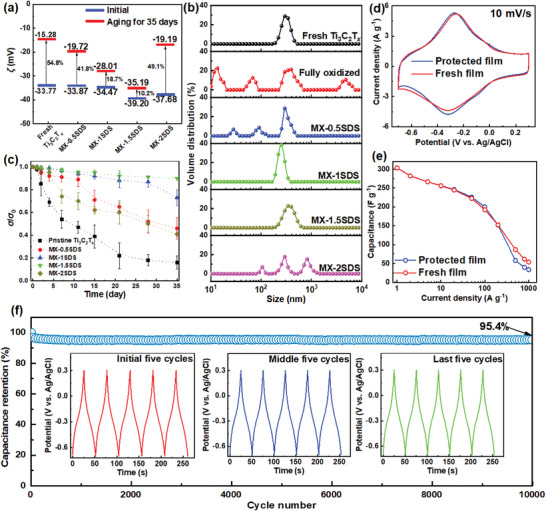
Dynamic light scattering analyses of Ti_3_C_2_T*_x_* suspensions before and after 35 days of aging with different SDS concentrations: evolution of a) *ζ* potentials and b) particle distributions; conductivity attenuation of Ti_3_C_2_T*_x_* films without and with SDS in c) different concentrations; d) cyclic voltammetry curves at 10 mV s^−1^ and e) rate capabilities of the MX‐1.5SDS and fresh MXene films; f) cycle performance of the MX‐1.5SDS film at 10 A g^−1^ with the charge/discharge profiles during the initial, middle, and last five cycles in the inset.

Adsorption and edge capping of SDS over Ti_3_C_2_T*_x_* was further verified by FTIR analysis of MX‐1.5SDS as shown in Figure S9 (Supporting Information). Typical bands with additional adsorption at 1125 cm^−1^ indexed to the stretching vibration of —OSO_3_^−^ reveal the adsorption of SDS on Ti_3_C_2_T*_x_*.^[^ ^25^ ^]^ Though the sample was repeatedly rinsed during filtration, a few SDS molecules still intercalate among Ti_3_C_2_T*_x_* nanosheets. Moreover, the stable peaks at 546 cm^−1^, corresponding to Ti—O bending, in the fresh and protected samples further confirm the oxidation resistance of MX‐1.5SDS. The variations of *ζ* potential (**Figure** **4**a) are recorded for Ti_3_C_2_T*_x_* dispersions after 35 days of aging with different SDS contents to understand the influence of SDS adsorption on the electric properties of Ti_3_C_2_T*_x_*. For MXene dispersion without SDS, *ζ* potential increases from −33.77 to −15.28 mV during 35 days (54.8% of attenuation). The *ζ* potential is steadily stabilized as SDS content rises to 1.5 mg mL^−1^ (−35.19 mV, 10.2% of attenuation). However, with a dose of 2 mg mL^−1^ (MX‐2SDS), an aggravated *ζ* potential attenuation (40.1%) occurs due to inadequate protection by surfactant micelle. Concomitantly, MX‐1.5SDS exhibits a particle size distribution similar to that of fresh Ti_3_C_2_T*_x_*, while other dispersions fail to achieve homogeneous particle distribution (Figure 4b) except for MX‐1SDS. This again supports the superiority of 1.5 mg mL^−1^ as the most suitable SDS content for stabilizing Ti_3_C_2_T*_x_* nanosheets.

The electrical conductivity is crucial in assessing the effect of SDS adsorption on the electrochemical activities of MXene. Derived from the absolute conductivity (Figure S10a, Supporting Information), Figure 4c summarizes the variation of normalized conductivity (*σ*/*σ*_0_) for Ti_3_C_2_T*_x_* films vacuum‐filtered from different dispersions with varied SDS contents. The rapid decline of *σ*/*σ*_0_ for pristine Ti_3_C_2_T*_x_* during 35 days of aging can be ascribed to the semiconductor property of the formed TiO_2_ and the Joule heat effect resulted from the aggravated electron collision by collapsed Ti—C backbone.^[^ ^26^ ^]^ In contrast, SDS‐protected samples present much slower falling in *σ*/*σ*_0_ over time. MX‐1.5SDS film holds the minimized decrease in conductivity over prolonged aging owing to the trace oxidation of Ti_3_C_2_T*_x_* boundary. However, raising SDS content to 2 mg mL^−1^ causes a significant drop in conductivity because of the observed oxidation. Also, Figure S10b (Supporting Information) shows the pH evolutions of Ti_3_C_2_T*_x_* dispersions with various SDS concentrations. For the alkalinity of SDS, a slight rise in the intrinsic pH for dispersions could be anticipated. In the initial five days, pH values of fresh Ti_3_C_2_T*_x_* and MX‐0.5SDS dispersions decrease rapidly, indicating accelerated MXene degradation. By comparison, MX‐1.5SDS dispersion exhibits relatively steady pH evolution that reveals a constant ionization balance throughout aging. The slight pH drift for MX‐1.5SDS may be ascribed to the uptake of hydroxyl ions by Ti_3_C_2_T*_x_* nanosheets.^[^ ^15c^ ^]^

The electrochemical performance of optimally stored Ti_3_C_2_T*_x_* dispersion was checked by direct utilizing the vaccum‐filtered MX‐1.5SDS film (from 35 days aged dispersion) as an electrode for supercapacitor in a three‐electrode system with 3 m sulfuric acid (H_2_SO_4_) electrolyte. Figure 4d illustrates the cyclic voltammetry (CV) curves of MX‐1.5SDS and fresh Ti_3_C_2_T*_x_* films at a scan rate of 10 mV s^−1^ in the potential of −0.7–0.3 V versus Ag/AgCl, where a redox couple is detected at ≈−0.32 V (oxidation) and −0.27 V (reduction), signifying their pseudocapacitive energy storage behavior. Almost overlapped CV curves of fresh MXene and MX‐1.5SDS films indicate that Ti_3_C_2_T*_x_* nanosheets are well‐preserved in water with 1.5 mg mL^−1^ SDS without losing their fundamental energy‐storage features. Figure 4e shows the specific capacitances of MX‐1.5SDS and fresh Ti_3_C_2_T*_x_* films at different current densities with only a trivial capacity difference at high current densities. Figure 4f depicts the long‐term cyclic performance of MX‐1.5SDS film at 10 A g^−1^, which possesses a capacitance retention of 95.4% after 10000 cycles. The charge/discharge curves in the initial, middle, and last five cycles also show similar profiles with negligible deformation, implying its excellent cycling durability and reversibility. Capacitive performance is also shown to be unaffected by the small amount of SDS left behind after a water‐based rinsing.

Multiscale simulations were conducted to elucidate the possible interaction between SDS^−^ and Ti_3_C_2_T*_x_*. MC and MD algorithms were used to screen SDS^−^ adsorption conformations on group‐free and defective MXenes. Figures S12 (MC) and S14 (MD) (Supporting Information) show an evenly distributed SDS^−^ over Ti_3_C_2_ surface. In contrast, a higher probability density of SDS^−^ could be observed for MXene with defective sites based on the lowest interaction energies (*E*_inter_), charge compatibility between the SDS^−^ and positively charged MXene defects. Authorized temperature and energy equilibriums for MD courses are displayed in Figure S13 (Supporting Information). The negative *E*_inter_ reveals SDS's adsorption spontaneity and affinity toward Ti_3_C_2_T*_x_* plane, which coincides well with the experimental conclusion that SDS could protect the oxidation‐sensitive regions. Guided by the screened conformations, adsorption processes of SDS on Ti_3_C_2_T*_x_* surfaces were studied by first‐principles DFT computation. Using SDS^−^ axis as a reference (Figure S15a, Supporting Information), three representative locations were selected for adsorption (Figure S15b, Supporting Information). All energy‐optimized conformations were deduced to follow route II, and thus this path was further used to explore DFT descriptors. Figure S16 (Supporting Information) illustrates the side and top views of SDS^−^ equilibrium conformations on bare and defective Ti_3_C_2_T*_x_* with the details of interacting sites, and adsorption energy (*E*_ads_) and height (*h*_ads_) are listed in Table S3 (Supporting Information). Strong anchoring of SDS on Ti_3_C_2_ surface is established in Figure S16a_1_ (Supporting Information) due to large *E*_ads_ value (−4.61 eV), verifying the spontaneous and stable adsorption. Similar interactions are observed in Figure S16b_1_–d_1_ (Supporting Information) for the adsorption of SDS on defective Ti_3_C_2_O_2_, Ti_3_C_2_F_2_, and Ti_3_C_2_(OH)_2_, yielding *E*_ads_ values of −3.09, −3.17, and −1.72 eV, respectively. Especially, steric hindrance of —OH terminals inevitably separates SDS from the contact with Ti atoms, and thus possible hydrogen bonds account for the stabilized adsorbate and relatively low *E*_ads_ modulus.

**Figure 5:**
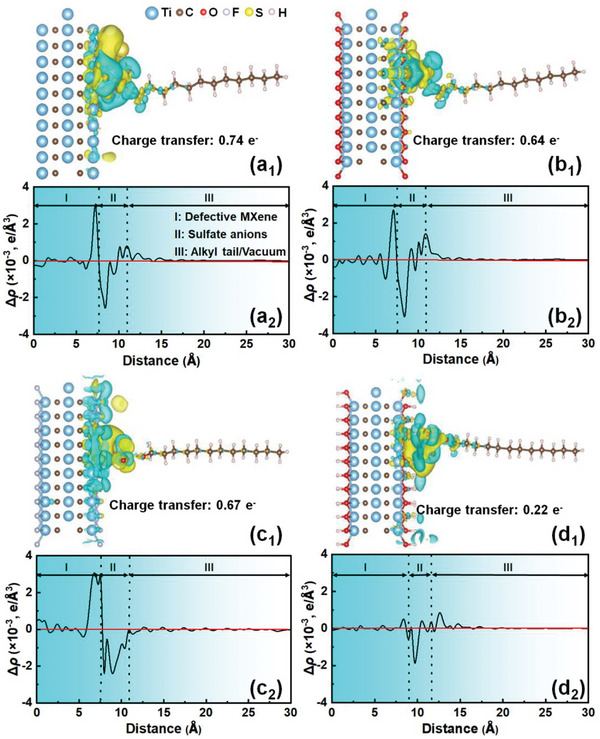
Charge density difference analyses (subscript 1) on the adsorption of SDS on a) group‐free Ti_3_C_2_ and b) defective Ti_3_C_2_O_2_, c) Ti_3_C_2_F_2_ and d) Ti_3_C_2_(OH)_2_ surfaces (isosurface: 0.005 e/Bohr^3^) accompanied by the corresponding planar‐average charge density difference (Δ*ρ*, subscript 2) along the *z*‐direction.

**Figure** **5** shows the possible electron transportation between SDS^−^ and Ti_3_C_2_T*_x_* based on the secondary charge density difference, where yellow and cyan colors in Figure 5a1–d1 represent the electron accumulation and depletion, respectively. Intensive electron exchange occurs primarily along the interface, specifically at defective regions of Ti_3_C_2_T*_x_* surfaces. This further confirms the developed modest interaction (e.g., electrostatic force) between SDS and Ti_3_C_2_T*_x_* surfaces that allows the physicochemical performances of MXene to be maintained after facile rinsing of SDS‐capped Ti_3_C_2_T*_x_*.^[^ ^27^ ^]^ The difference in planar‐average charge density (Figure 5a2–d2) also supports the interfacial electron transportation for the intense charge buildup (peak) and depletion (valley). Hence, it can be assumed that SDS behaves as an electron donor to Ti_3_C_2_T*_x_* surfaces via the negatively charged sulfate terminal. Bader charge analysis also reveals the smaller charge transfer at the defective site Ti_3_C_2_(OH)_2_ (0.22 e^−^) compared to other analogues, suggesting the inferior binding strength of SDS. The largest charge transfer (0.74 e^−^) calculated for the Ti_3_C_2_ system stems from the positive surface electrostatic potential of Ti_3_C_2_ as shown in Figure S17a (Supporting Information). Likewise, defective sites and partial edges on Ti_3_C_2_O_2_ (Figure S17b_2_, Supporting Information) and Ti_3_C_2_F_2_ (Figure S17c_2_, Supporting Information) holding positive charge preferentially attract the sulfate group on SDS. Consequently, slightly more electrons are transported from SDS to defective Ti_3_C_2_F_2_ (0.67 e^−^) than Ti_3_C_2_O_2_ (0.64 e^−^) by virtue of the greater electronegativity of fluorine (3.98 vs. 3.44 for oxygen) atoms.^[^ ^28^ ^]^ As per the reported MXene oxidation mechanism,^[^ ^7^ ^]^ the electron transfer motivated by the formation of the internal electric field between Ti cations and nearby C^4−^ species is effectively inhibited in the presence of SDS through electron donation of the sulfate terminal, which in turn retards the progression of Ti_3_C_2_T*_x_* degradation.

## 3 Conclusion

In summary, we report a simple, but efficient strategy to stabilize Ti_3_C_2_T*_x_* aqueous colloid using SDS as an anti‐oxidant. At an optimal dosage of 1.5 mg mL^−1^, SDS could efficiently protect oxidation‐susceptible sites on Ti_3_C_2_T*_x_* nanosheets, which enabled MX‐1.5SDS to achieve maximum oxidation resistance and excellent dispersity, allowing for up to 213 days of colloidal stability. The concentration‐dependent anti‐oxidation capability of SDS for Ti_3_C_2_T*_x_* aqueous dispersions was illustrated, which followed an increasing order with rising SDS content to 1.5 mg mL^−1^, while the sample with 2 mg mL^−1^ SDS suffered serious oxidation due to the formation of surfactant micelle. Moreover, since color variation is unmistakable in defining Ti_3_C_2_T*_x_* oxidation, a chroma strategy is, for the first time, established for the well‐suited measurement of the oxidation degree of MXenes. The anti‐oxidation mechanism of SDS was further investigated by MC and MD simulations, as well as DFT calculations. The secondary charge density difference modulated for electron transportation between SDS and Ti_3_C_2_T*_x_* surface supported the adsorption of SDS's sulfate terminals with excess electrons on the positively‐charged defects for the charge compatibility principle, while the alkyl chains prevented water and dissolved oxygen from approaching nanosheets. Although the evidenced adverse effect on certain applications (e.g., inkjet printing) for MXene with surfacants, the employed protection strategy exhibited negligible influence on capacitive property of the vacuum‐filtered film. This study bridges the gap between theoretical and experimental work for ionic stabilization of 2D MXenes, with hopeful implications for their long‐term colloidal storage without oxidation and compromising physicochemical potential.

## 4 Experimental Section

### Materials Synthesis: Materials

Ti_3_AlC_2_ was procured from Jilin 11 Technology Co., Ltd. (China); sodium dodecyl sulfate (SDS) of the guaranteed grade was supplied by Beijing Innochem Company (China), whose critical micelle concentration was examined through conductivity determination of the designed solutions with different dosages through a DZS 718 multi‐parameter analyzer (Shanghai INESA Scientific Instrument Co., Ltd., China). Deionized (DI) water from a ZYpureEDIA‐100‐UP system (Beijing Zhongyang Yongkang Environmental Science Co. Ltd, China) was boiled, naturally cooled and standing for 48 h before MXene dispersions preparation.

### Materials Synthesis: Synthesis of Ti_3_C_2_T*_x_* MXene Dispersion

The Ti_3_C_2_T*_x_* MXene was prepared by selectively etching Ti_3_AlC_2_ precursor via LiF/HCl etchant. Briefly, 0.99 g of LiF was dissolved in 10 mL of HCl (12 m) to form a homogeneous solution. Ti_3_AlC_2_ (1 g) powders were decanted into the LiF/HCl mixture and stirred for 24 h at 35 °C to achieve the etching of Al layers from Ti_3_AlC_2_. Afterward, the product was repeatedly washed with DI water and centrifugated at 5200 rpm for 5 min to remove the supernatant until its pH reached 7. The sediment was then re‐dispersed in DI water and ultrasonicated at 240 W for 30 min to realize the exfoliation of multilayer Ti_3_C_2_T*_x_*. After centrifugation at 5200 rpm for 1 h, the single‐layered MXene dispersion was obtained by collecting the black‐green supernatant. The concentration of MXene in the dispersion was estimated by weighting a vacuum‐filtered film (dried in a vacuum oven at 90°C) from the MXene dispersion with a known volume.

### Anti‐Oxidation Experiments for Ti_3_C_2_T*_x_*

Concentrated Ti_3_C_2_T*_x_* dispersions were diluted to 0.05 mg mL^−1^ by DI water, mixed with different amounts of SDS yielding the varied dosage of 0.5, 1.0, 1.5, and 2.0 mg mL^−1^, filled in glass vials in batches, and henceforth labeled as MX‐0.5SDS, MX‐1SDS, MX‐1.5SDS, and MX‐2SDS, respectively. The fresh Ti_3_C_2_T*_x_* dispersion (0.5 mg mL^−1^) without SDS was set as control, which underwent the natural oxidation process. Importantly, non‐hermetic vials were placed in a dark cage for aging to avoid the evident effect of light.^[^ ^9b^ ^]^ The protected and unprotected Ti_3_C_2_T*_x_* dispersions were all placed at the lab‐ambient conditions.

### Characterizations of the Anti‐Oxidative Effect

Macroscopic appearances of pristine Ti_3_C_2_T*_x_*, MX‐0.5SDS, MX‐1SDS, MX‐1.5SDS, and MX‐2SDS dispersions were recorded by an EOS 850D digital camera (Canon, Japan) after allocated aging intervals (0, 1, 2, 4, 7, 11, 15, 21, 28, and 35 days). Accordingly, ultraviolet–visible (UV–vis) spectra of the corresponding dispersions were recorded at a wavelength of 200–800 nm after the aforementioned intervals in the quartz cuvette of a 10 mm optical path via a UV‐2450 spectrometer (Shimadzu, Japan) taking DI water as the reference. For the first time, the total chroma (ΔE* ab) strategy was employed to evaluate the degradation process of Ti_3_C_2_T*_x_* dispersions without and with different concentrations of SDS. An LC100 colorimeter (Lovibond, United Kingdom) was used to analyze ΔE* ab value of different dispersions, which is defined as follows:

(3) $$\Delta E_{\mathrm{ab}}^{*}=\sqrt{{\left(\Delta L\right)}^{2}+{\left(\Delta a\right)}^{2}+{\left(\Delta b\right)}^{2}}$$

where Δ*L*, Δ*a*, and Δ*b* denote the couple contributions of light/shade, red/green, and yellow/blue, respectively. Notably, each Ti_3_C_2_T*_x_* dispersion volume was on‐demand replenished by DI water before chroma determinations.

After each aging period, unprotected and protected Ti_3_C_2_T*_x_* dispersions were vacuum‐filtered and carefully rinsed with DI water, followed by the analyses of X‐ray photoelectron spectroscopy (XPS, ESCALAB 250, Thermo Scientific, United States), field emission scanning electron microscopy (SEM, Quanta FEG 250, FEI, United States) and X‐ray diffraction (XRD, Panalytical X'Pert powder, the Netherlands). In detail, XPS measurements were fulfilled with an Al K*α* radiation source (1486.6 eV) applying a step energy of 20 eV and pressure of 3 × 10^−8^ Pa. The obtained spectra were deconvoluted using Gaussian functions after subtracting a modified‐Shirley background. SEM images of different films were captured under 15 kV accelerating voltage and 700 nA probe current. The cross‐section morphologies of Ti_3_C_2_T*_x_* films were surveyed by fracturing under the uniaxial tension. XRD patterns for the films before and after 35 days of aging were collected using Cu K*α* radiation (*λ* = 0.154 nm) inspired at 40 kV and 30 mA, and each sample was scanned from 5° to 90° (2*θ*) with a step size of 0.04°. At each allocated aging interval, the unprotected and protected Ti_3_C_2_T*_x_* dispersions were vacuum‐filtered accompanied by several rinsing procedures, whose electronic conductivities in the dry state were determined by an ST‐2258C four‐point collinear probe (Suzhou Jingge Electronic Company, China). Each probe diameter of 80 µm and a distance of 1.6 mm between adjacent points were utilized. Fourier transform infrared spectra (FT‐IR) of fresh, fully oxidized Ti_3_C_2_T*_x_* and MX‐1.5SDS vacuum‐filtered films were recorded by a Nicolet iN10 instrument (Thermo Scientific, USA).

High‐resolution transmission electron microscopy (TEM, Talos 200s, FEI, USA) was employed to probe the oxidation resistance of pristine Ti_3_C_2_T*_x_*, MX‐0.5SDS, MX‐1SDS, MX‐1.5SDS, and MX‐2SDS dispersions after 35 days of aging, which were initially DI water‐diluted and drop‐cast on the copper grids (2000 mesh, Beijing Zhongxing Bairui Technology Co., Ltd). The Tyndall effect of the most stable colloidal dispersion was examined by an incident laser. The *ζ* potentials and average hydrodynamic diameters of different Ti_3_C_2_T*_x_* dispersions before and after aging were measured through dynamic light scattering realized by a BeNano 180 Zeta Pro instrument (Bettersize, China).

### Electrochemical Measurement

The electrochemical performance of MXene‐1.5SDS film after 35 days aging as the electrode of supercapacitors was measured to investigate the performance stability of protected MXene taking fresh Ti_3_C_2_T*_x_* film as a control. It should be highlighted that repeated flushing with DI water was conducted during filtering to eliminate the influence of introduced SDS. MXene‐1.5SDS and fresh Ti_3_C_2_T*_x_* films were cut into disks (diameter of 5 mm), and then directly used as electrodes for supercapacitors.

Electrochemical measurements were performed by assembling a three‐electrode system using greatly excessive activated carbon, Ag/AgCl, and 3 m H_2_SO_4_ solution as the counter electrode, reference electrode, and electrolyte, respectively. Cyclic voltammetry curves were measured on a Bio‐Logic electrochemical workstation (VSP) in the potential range of −0.7–0.3 V versus Ag/AgCl. The capacitances (*C*_g_) at various current densities were calculated from the galvanostatic charge/discharge curves accomplished on an Arbin BT2000 battery test equipment according to the following equation:

(4) $$C_{g}=\frac{It_{d}}{\Delta Vm}$$

where *I* is the current (A), *t*_d_ is the discharge time (s), Δ*V* is the potential window (V), and *m* is the mass of the working electrode (g).

### Computational Methods

Multi‐scale simulations were performed to reveal the underlying anti‐oxidative mechanism of SDS for Ti_3_C_2_T*_x_* aqueous dispersions. The structure of SDS was downloaded from “The Materials Project” website (https://www.materialsproject.org/). Monolayer Ti_3_C_2_T*_x_* (T: O, F, and OH) models with P‐3 M1 space group were constructed and enlarged to 4 × 4 × 1 supercell as the substrate. Given that the oxidation of MXene initiates from defects, especially the tailored edges, partial surface groups on the built models were manually removed to represent the surface flaws. Hence, the deficient charges adjacent to the defective area were imparted during energy calculations.

As a matter of priority, the energy favored adsorption conformation of SDS was explored in atomic‐scale Monto Carlo (MC) simulations via the adsorption locator module in Materials Studio software (BIOVIA, France). A vacuum layer of 25 Å was built over the Ti_3_C_2_T*_x_* substrate, in which one SDS anion (SDS^−^) and the counter cation (Na^+^) were placed randomly. The simulated annealing task was conducted for 20 cycles at 100000 steps per cycle with the controlled temperature from 10^5^ to 10^2^ K under a Universal forcefield. The feasibility of this forcefield had been verified for the interaction between MXenes and exogenous molecules.^[^ ^29^ ^]^ The interaction energy (*E*_inter_, kJ mol^−1^) derived from MC simulations was acquired through the following expression:^[^ ^30^ ^]^

(5) $$E_{\mathrm{inter}}=E_{\mathrm{tot}}-\left(E_{\mathrm{MXene}}+E_{\mathrm{SDS}}\right)$$

where *E*_tot_, *E*_MXene_, and *E*_SDS_ are the energies of the total system, Ti_3_C_2_T*_x_* substrate, and SDS, respectively.

Molecular dynamics (MD) simulation was conducted via Forcite plus module in succession to involve the solvent effect. The vacuum layer of 25 Å was built over each surface of different MXenes (i.e., bare Ti_3_C_2_, Ti_3_C_2_O_2_, Ti_3_C_2_F_2_, and Ti_3_C_2_(OH)_2_). 565 water molecules (1 g/cm^3^), one SDS^−^, and one Na^+^ were contained in each vacuum layer, in which SDS^−^, and Na^+^ were randomly arranged. Electrostatic and van der Waals interactions were dealt with Group‐based cutoff and Ewald schemes. After full geometry optimization using Universal forcefield, the Quench mode was performed at 298 K under NVT canonical ensemble (constant system atoms, volume and temperature modulated by Nose thermoset) for 10 ns with a step size of 1 fs. The last 100 equilibrium dynamic outcomes were averaged to obtain the value of *E*_inter_ as per the following expression:^[^ ^30^ ^]^

(6) $$E_{\mathrm{inter}}=E_{\mathrm{tot}}-\left(E_{\mathrm{MX}-\mathrm{sol}}+E_{\mathrm{SDS}}\right)$$

where *E*_MX‐sol_ is the energy of Ti_3_C_2_T*_x_* substrate and all water molecules; *E*_tot_ and *E*_SDS_ have the same meaning as those described for Equation 5.

Finally, first‐principles investigations based on density functional theory (DFT) were fulfilled through the Vienna ab initio simulation software package (VASP). Spin‐polarized DFT calculations were conducted using the exchange‐correlation interactions described by generalized gradient approximation (GGA) in the form of Perdew–Burke–Ernzerhof (PBE) functional. Valence electronic states were expanded in terms of plane waves with the core‐valence interaction represented by projector augmented plane wave (PAW) and a cutoff of 450 eV. A vacuum layer of 15 Å along the *z*‐direction was built on different Ti_3_C_2_T*_x_* models to avoid the mirror interaction between the adjacent supercells. The Brillouin zone integration was sampled using 2 × 2 × 1 *k*‐points mesh for geometry optimization, and 3 × 3 × 1 *k*‐points mesh for adsorption simulations. DFT‐D3 with the Grimme scheme was employed to correct the dispersion effect resulting from non‐bonding interactions. The adsorption energy (*E*_ads_, eV) of SDS on the MXene surface was calculated via the following equation:

(7) $$E_{\mathrm{ads}}=E_{\mathrm{tot}}-\left(E_{\mathrm{MX}}+E_{\mathrm{SDS}}\right)$$

where *E*_tot_ is the total energy of the system and *E*_MX_ and *E*_SDS_ are the energies of Ti_3_C_2_T*_x_* substrate and SDS, respectively.

## Conflict of Interest

The authors declare no conflict of interest.

## Supporting information

Supporting Information

## Data Availability

The data that support the findings of this study are available from the corresponding author upon reasonable request.
