# Supplementary material for: Effect of Sodium Dodecyl Sulfate on Stability of MXene Aqueous Dispersion
Source: Adv Sci (Weinh). 2023 Jun 22;10(24):2300273. doi: 10.1002/advs.202300273 (PMC10460840; doi:10.1002/advs.202300273)
Supplement: Supplementary file 1 — Supporting Information [file ADVS-10-2300273-s001.pdf]

ADVANCED SCIENCE
Open Access

Supporting Information

for *Adv. Sci.*, DOI 10.1002/advs.202300273

Effect of Sodium Dodecyl Sulfate on Stability of MXene Aqueous Dispersion

*Baomin Fan, Xiaoqi Zhao, Peng Zhang, Yi Wei, Ning Qiao, Biao Yang, Razium A. Soomro, Ran Zhang and Bin Xu**

Supporting Information

Effect of Sodium Dodecyl Sulfate on Stability of MXene Aqueous Dispersion

*Baomin Fan, Xiaoqi Zhao, Peng Zhang, Yi Wei, Ning Qiao, Biao Yang, Razium A. Soomro, Ran Zhang, and Bin Xu**

Dr. B. Fan, X. Zhao, B. Yang
College of Chemical and Materials Engineering
Beijing Technology and Business University
Beijing, 100048 China

Dr. P. Zhang, Y. Wei, Dr. N. Qiao, Dr. R. A. Soomro, R. Zhang, Prof. B. Xu
State Key Laboratory of Organic-Inorganic Composites
Beijing Key Laboratory of Electrochemical Process and Technology for Materials
Beijing University of Chemical Technology
Beijing, 100029 China
E-mail: xubin@buct.edu.cn, binxumail@163.com

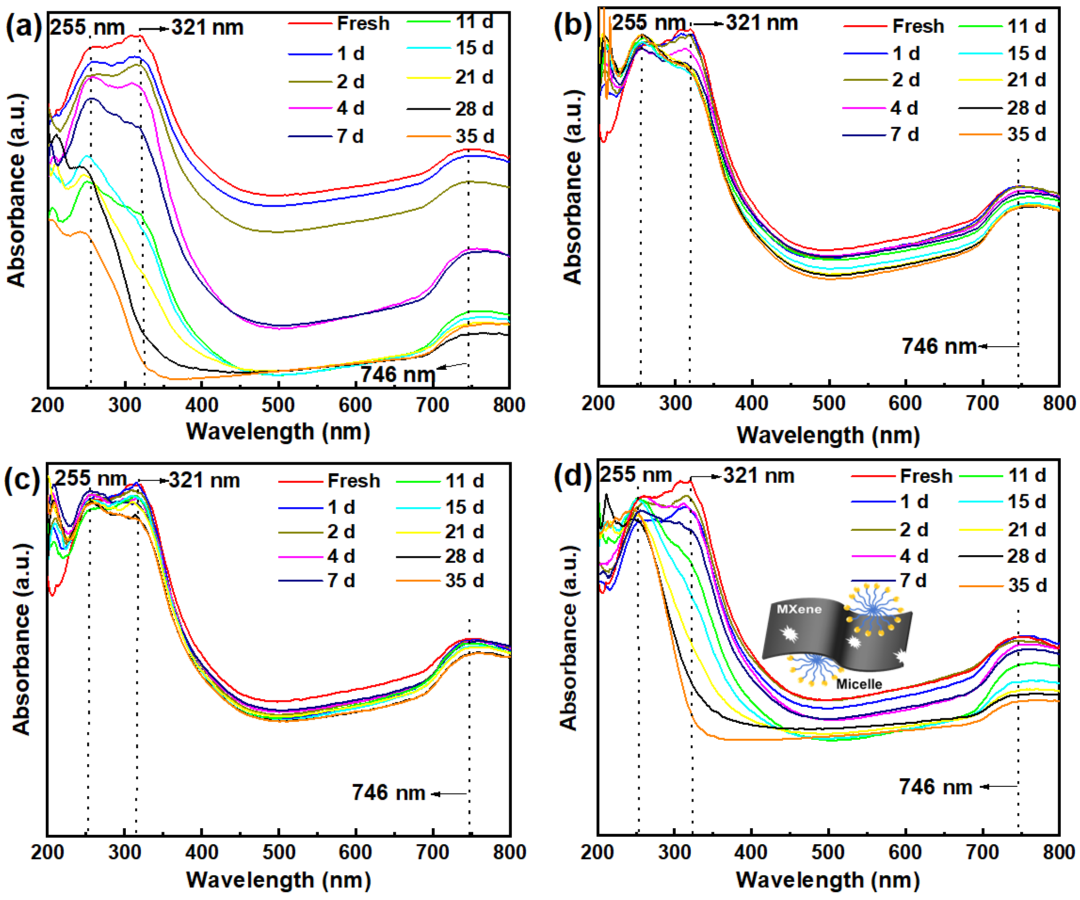

Figure S1 UV-vis spectra of $Ti_3C_2T_x$ suspensions without (a) and with 0.5 (b), 1.0 (c) and 2.0 mg/mL (d) SDS after the allocated aging period.

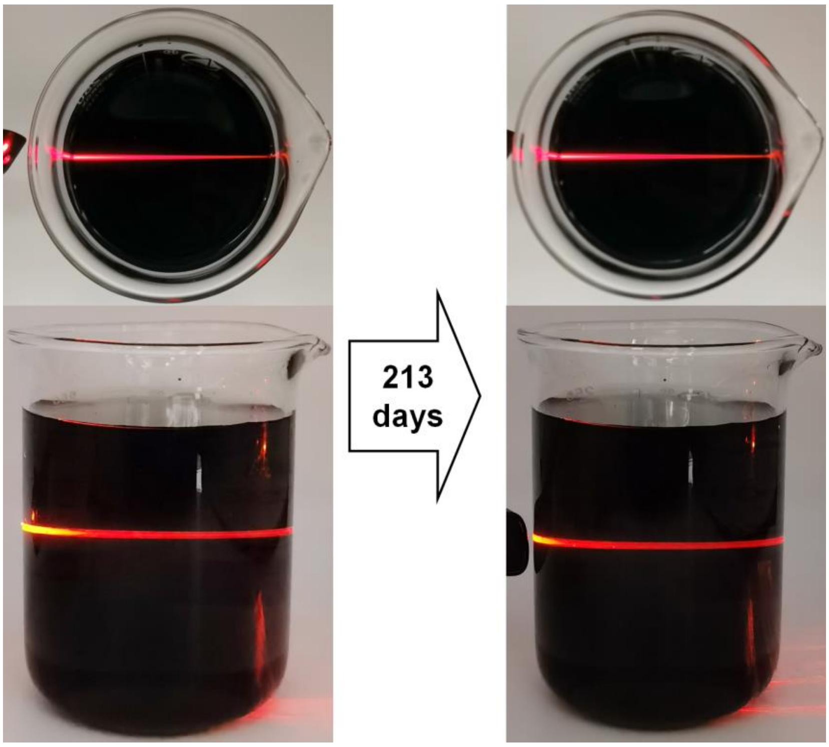

Figure S2 Tyndall effect of MX-1.5SDS sample before and after 213 days of aging (solution volume: 250 mL).

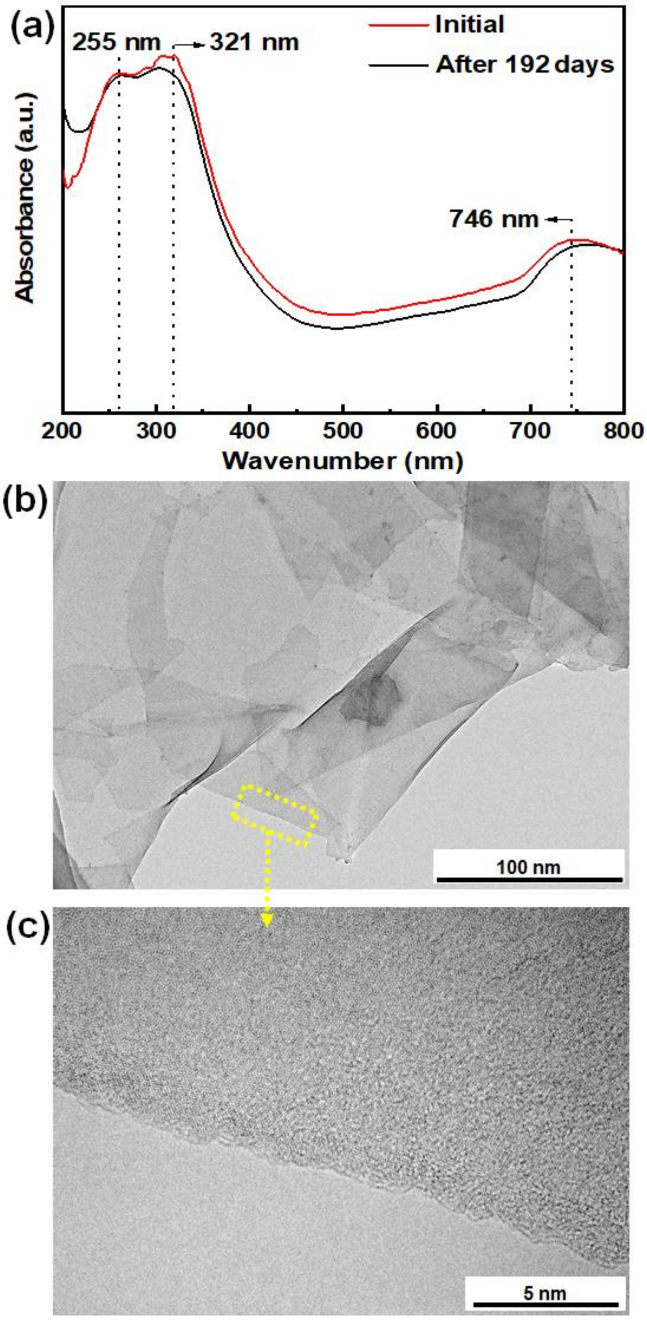

Figure S3 UV-vis spectra of fresh and protected (213 days, with 1.5 mg/mL SDS) $Ti_3C_2T_x$ dispersions (a); TEM image of the protected $Ti_3C_2T_x$ nanosheet (b) together with the high-resolution image spotted on the edge.

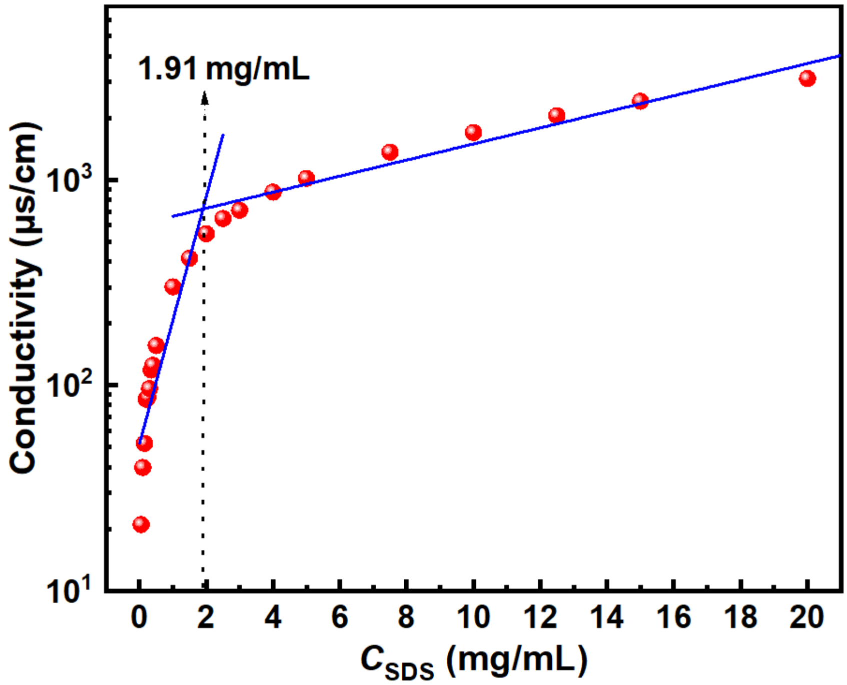

Figure S4 Critical micelle concentration (CMC) evaluation of utilized SDS through conductivity determinations at 298 K.

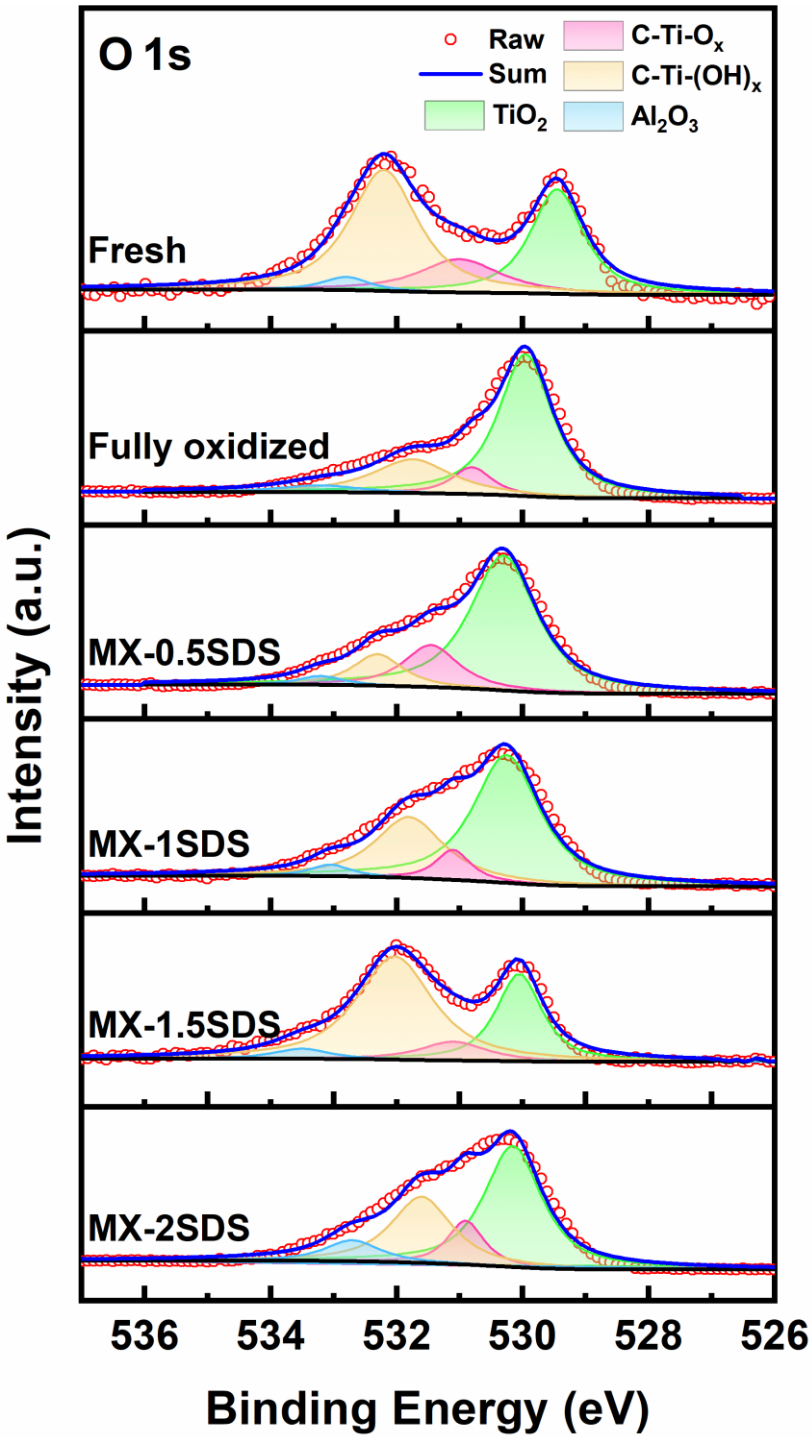

Figure S5 High-resolution XPS spectra of O 1s for fresh, fully oxidized $Ti_3C_2T_x$ film, and the SDS protected films with dosage of SDS after 35 days of aging.

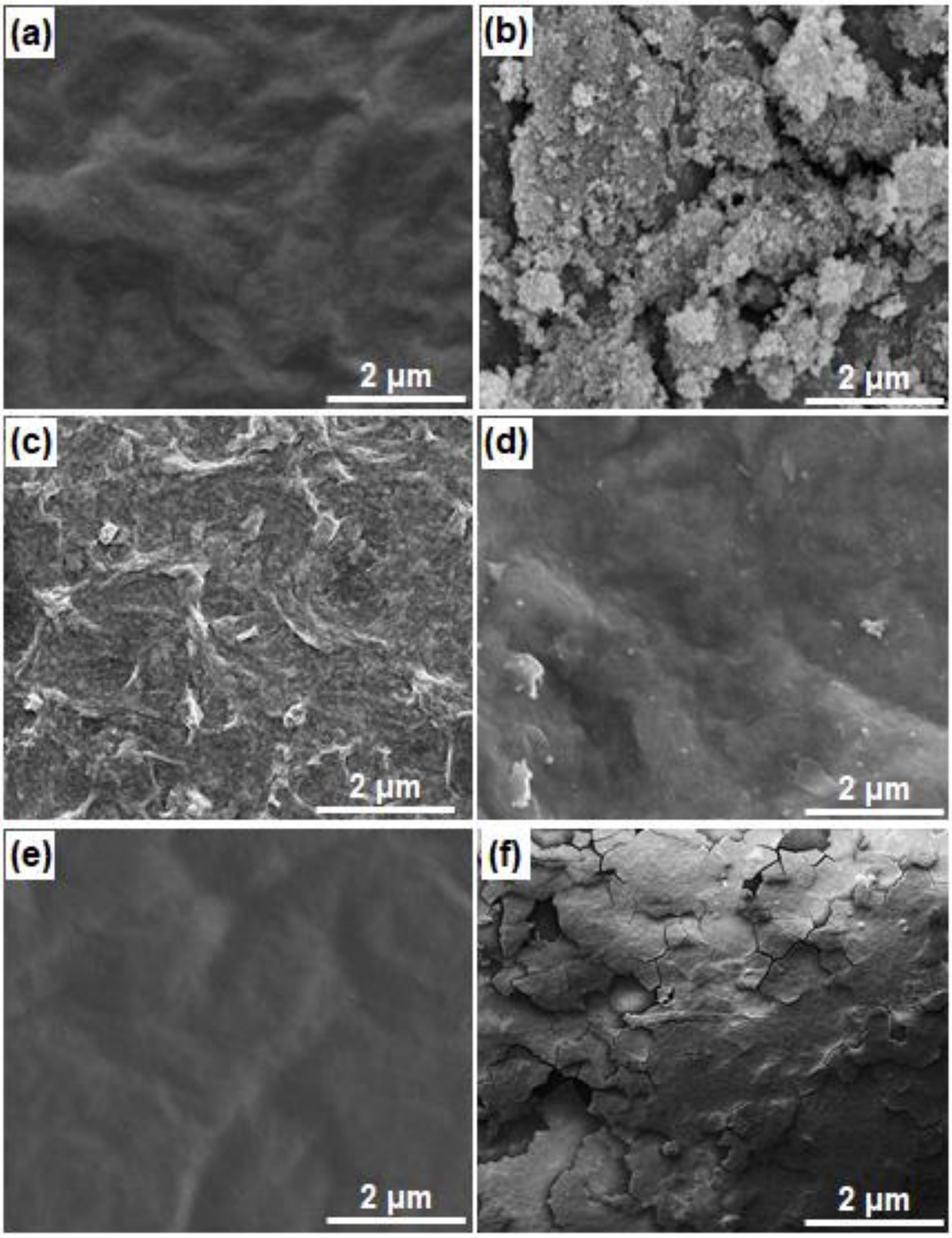

Figure S6 Surface morphologies of the vacuum-filtered $Ti_3C_2T_x$ films from freshly etched (a), unprotected (b) and protected $Ti_3C_2T_x$ solutions with 0.5 (c), 1.0 (d), 1.5 (e) and 2.0 (f) mg/mL SDS after 35 days of aging.

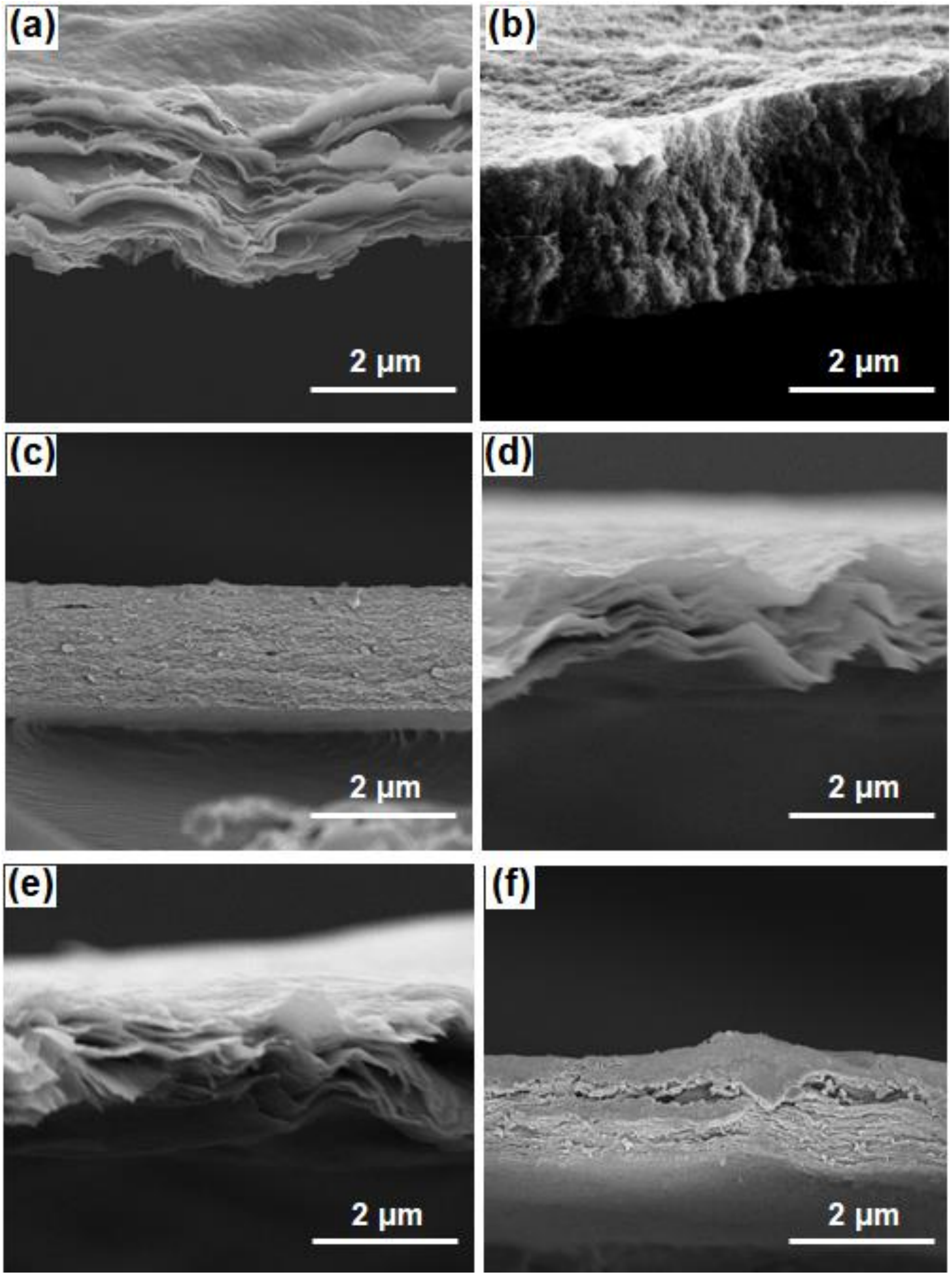

Figure S7 Cross-section images of the vacuum-filtered $Ti_3C_2T_x$ films from freshly etched (a), unprotected (b) and protected $Ti_3C_2T_x$ solutions with 0.5 (c), 1.0 (d), 1.5 (e) and 2.0 (f) mg/mL SDS after 35 days of aging.

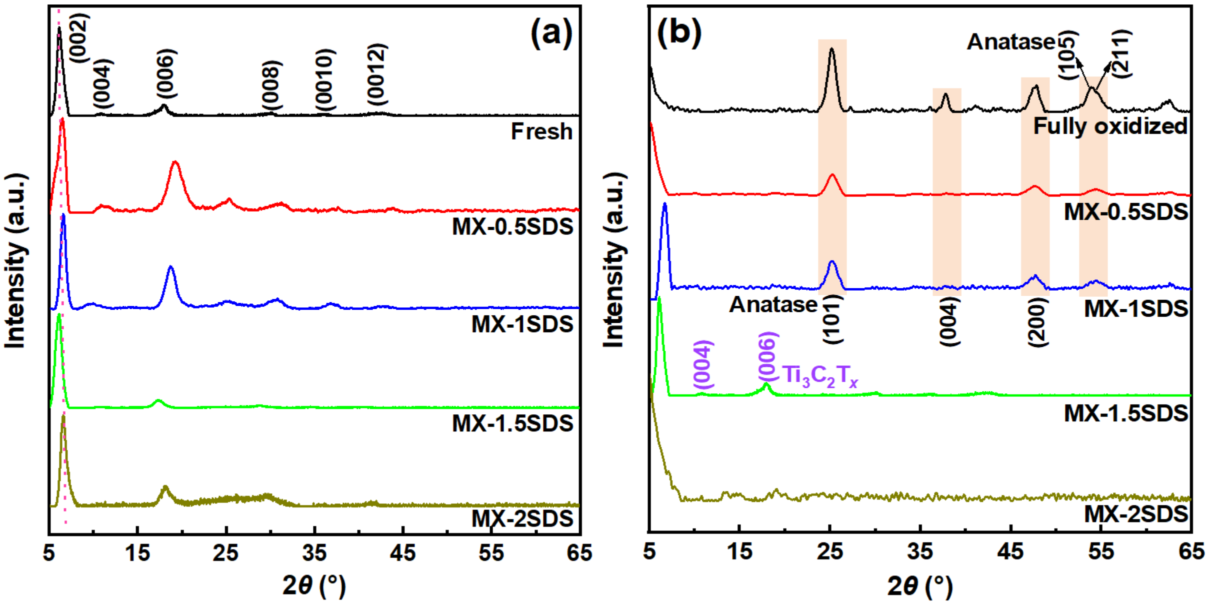

Figure S8 XRD patterns of vacuum-filtered $Ti_3C_2T_x$ films before (a) and after 35 days of aging (b) protected by different concentrations of SDS.

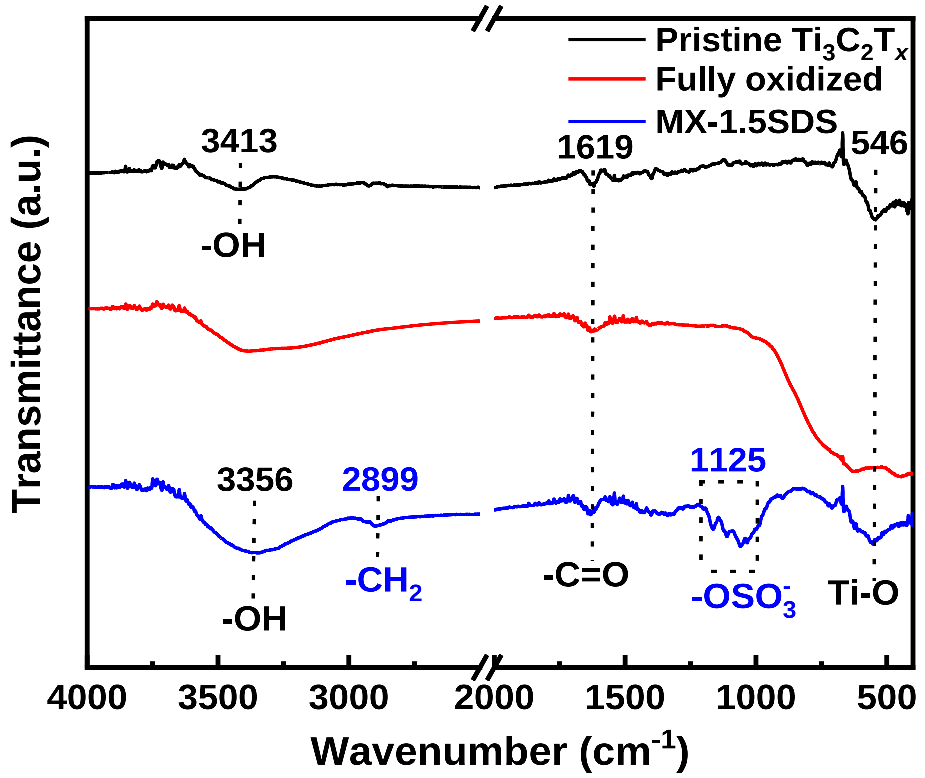

Figure S9 FTIR spectra of the pristine $Ti_3C_2T_x$, unprotected sample and the sample protected by 1.5 mg/mL SDS after 35 days of aging.

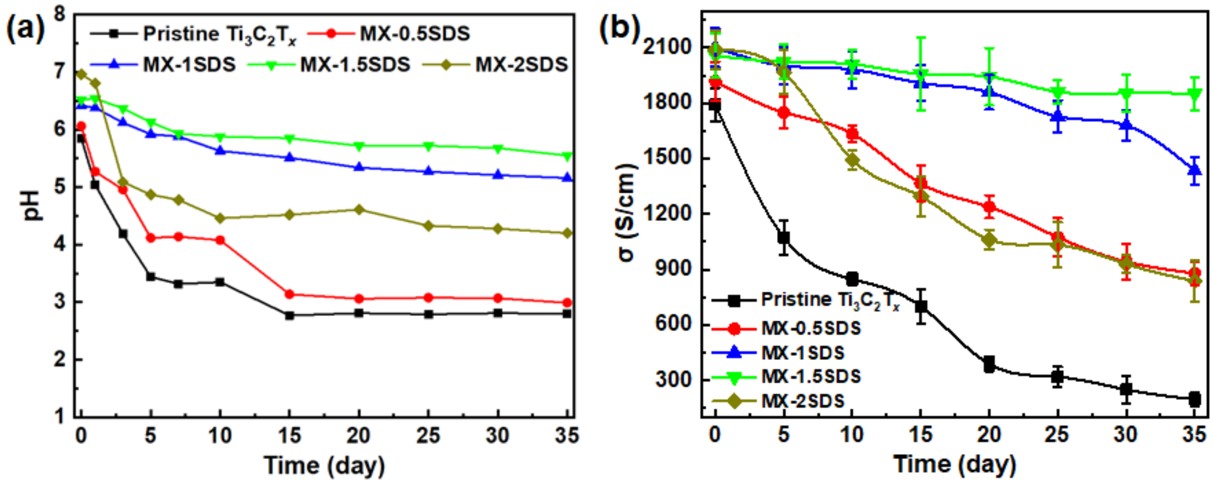

Figure S10 pH evolutions (a) of $Ti_3C_2T_x$ dispersions and their corresponding film conductivities (b) with different SDS concentrations at 298 K.

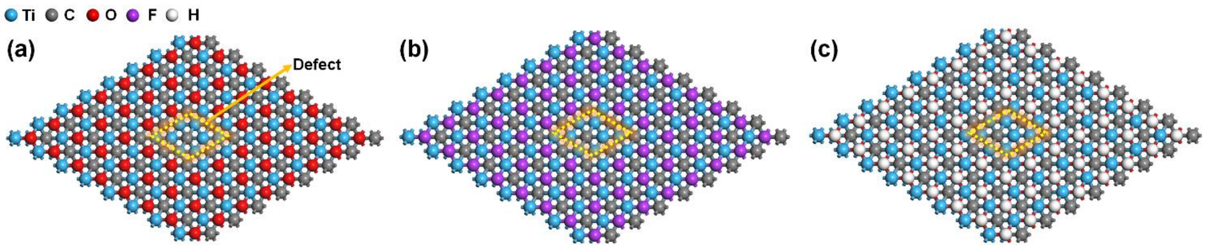

Figure S11 Top snapshots of $Ti_3C_2O_2$ (a), $Ti_3C_2F_2$ (b) and $Ti_3C_2(OH)_2$ (c) with fabricated defects.

Figure S11a, S11b and S11c displays the manufactured defects (yellow framed) on $Ti_3C_2O_2$, $Ti_3C_2F_2$ and $Ti_3C_2(OH)_2$, respectively, which are subsequently utilized in MC and MD simulations.

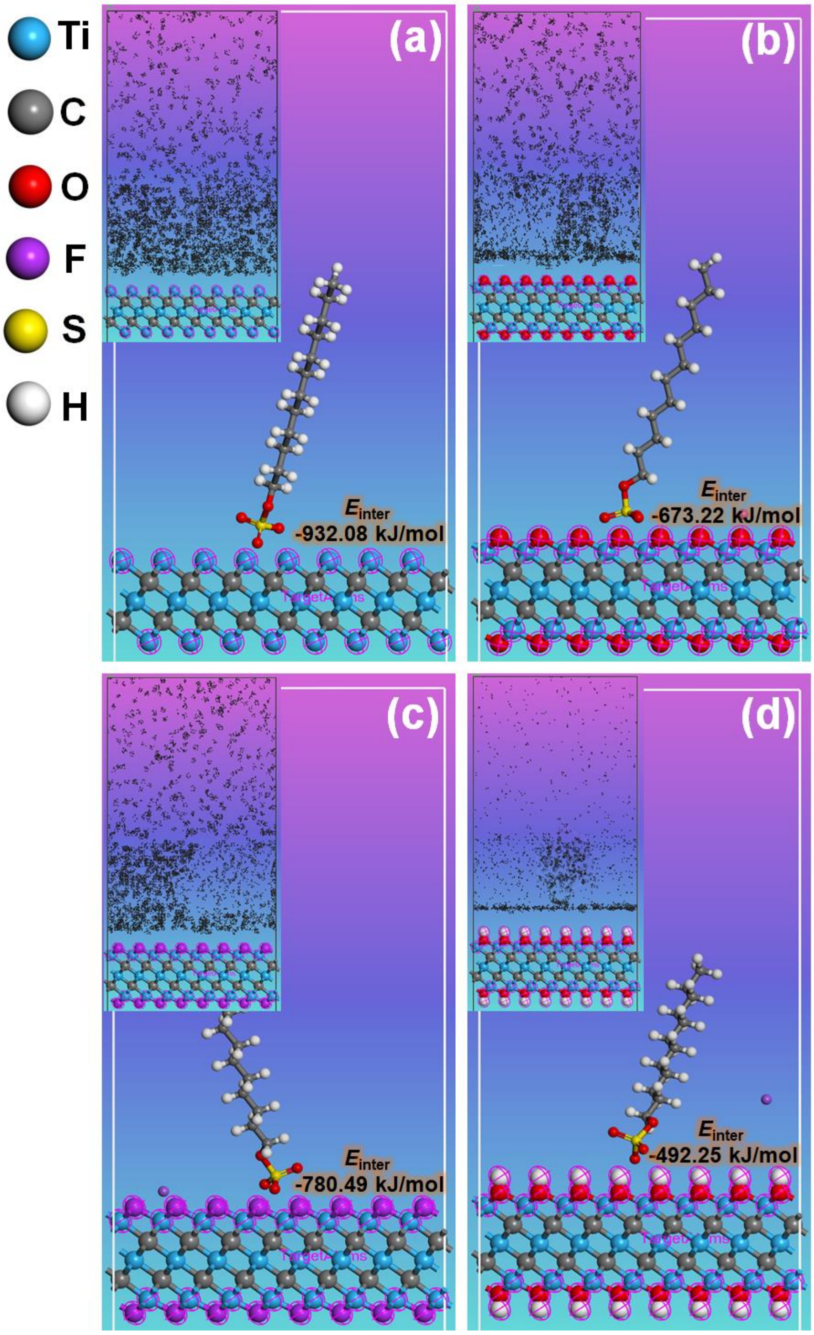

Figure S12 Adsorption conformation of SDS on pristine Ti_3C_2 (a), defective $Ti_3C_2O_2$ (b), $Ti_3C_2F_2$ (c) and s $Ti_3C_2(OH)_2$ surfaces along with the density profiles of SDS anion (inset).

In Figure S12a, the sulfate terminal on SDS favors contact with Ti_3C_2 substrate accompanied by the substantial probability around the surface (shown in the inset). Likewise, resembled adsorption manner is observed in Figure S12b-d for SDS on defective $Ti_3C_2T_x$ surfaces, which primarily occurs on the fabricated defects for the high possibility (black dots) in the density profiles.

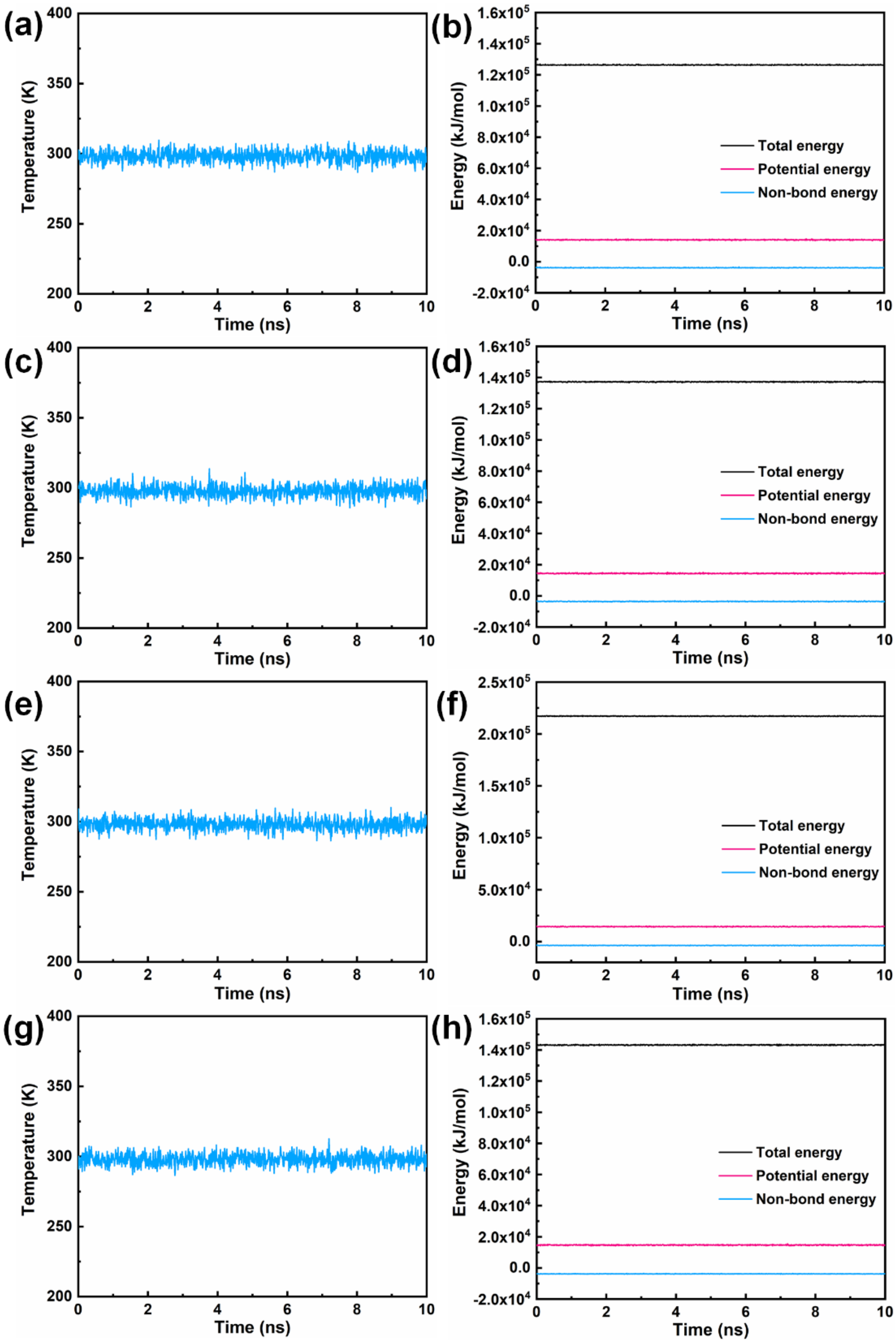

Figure S13 Temperature (left) and energies (right) fluctuations during molecular dynamics relaxation of SDS-stabilized MXene systems: (a, b) Ti_3C_2, (c, d) $Ti_3C_2O_2$, (e, f) $Ti_3C_2F_2$ and (g, h) $Ti_3C_2(OH)_2$.

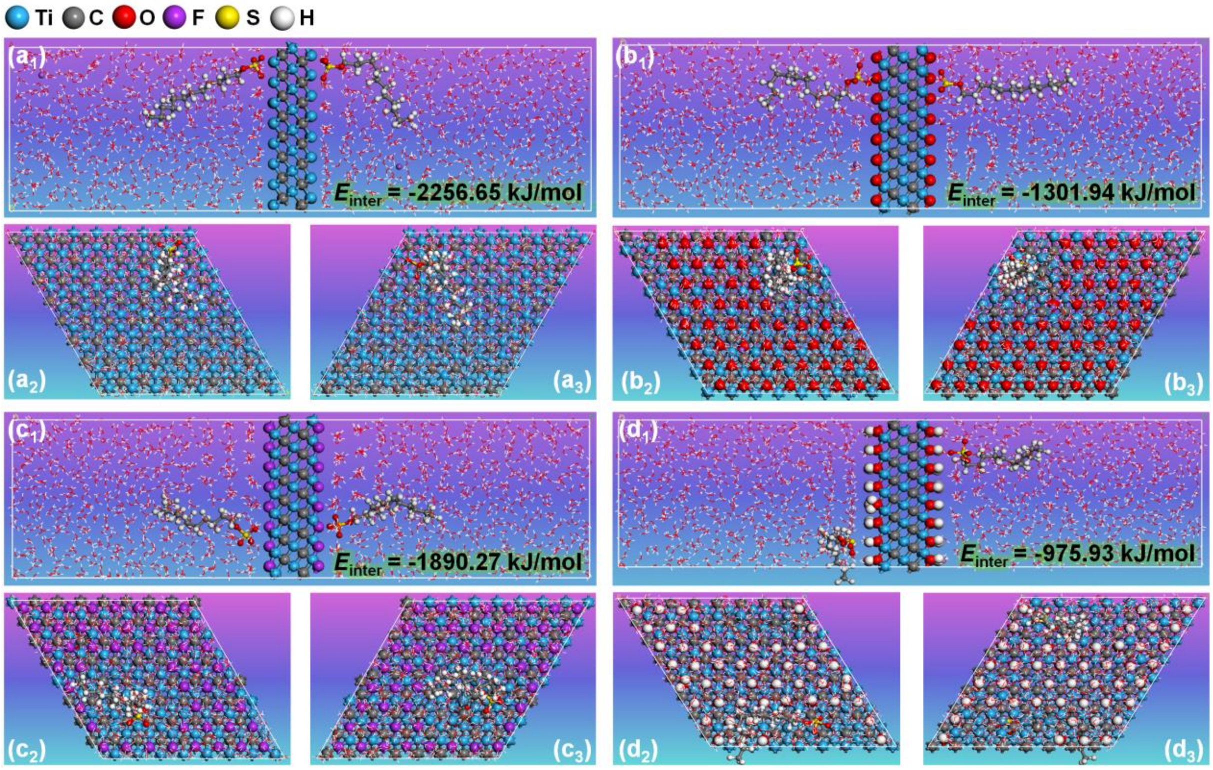

Figure S14 Molecular dynamic outcomes for the adsorption of SDS on defective Ti_3C_2 (a), $Ti_3C_2O_2$ (b), $Ti_3C_2F_2$ (c) and $Ti_3C_2(OH)_2$ (d); subscript 1: full view of the equilibrium conformation; subscript 2: left side view; and subscript 3: right side view.

Figure S13 provides the fluctuations of temperature and energies during the dynamic relaxation for the adsorption course of SDS on the $Ti_3C_2T_x$ surface. Notably, little bonding sign discerned by experimental assays validates the availability of a non-reactive Universal forcefield in this investigation. To involve the solvent (water) effect, MD simulations were conducted, and the outcomes are displayed in Figure S14. For both atomic-scale simulations, similar orientations occurred for SDS adsorbed on $Ti_3C_2T_x$ surfaces that the sulfate terminal tends to deposit on bare or defective sites leaving the alkyl chain outward. The obtained adsorption propensity consolidates the dominant Colombian force for SDS^- to recognize oxidation-sensitive centers of $Ti_3C_2T_x$. Owing to the absence of capping terminals, the strongest interaction is gained between SDS and Ti_3C_2 surface with the highest moduli of E_{inter} in both MC and MD simulations.

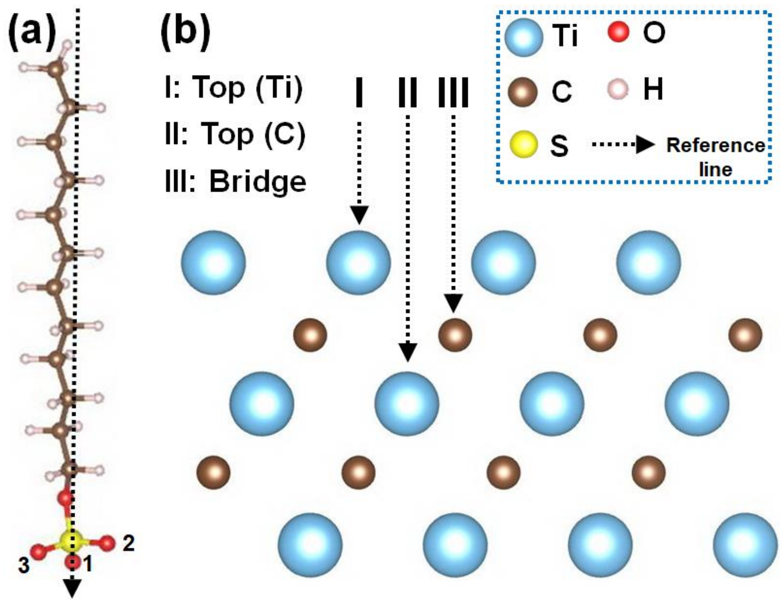

Figure S15 Adsorption sites (b) selected on different MXene surfaces as per the reference line on SDS (a, number 1-3 is utilized to locate the specific interaction sites, *vide infra*).

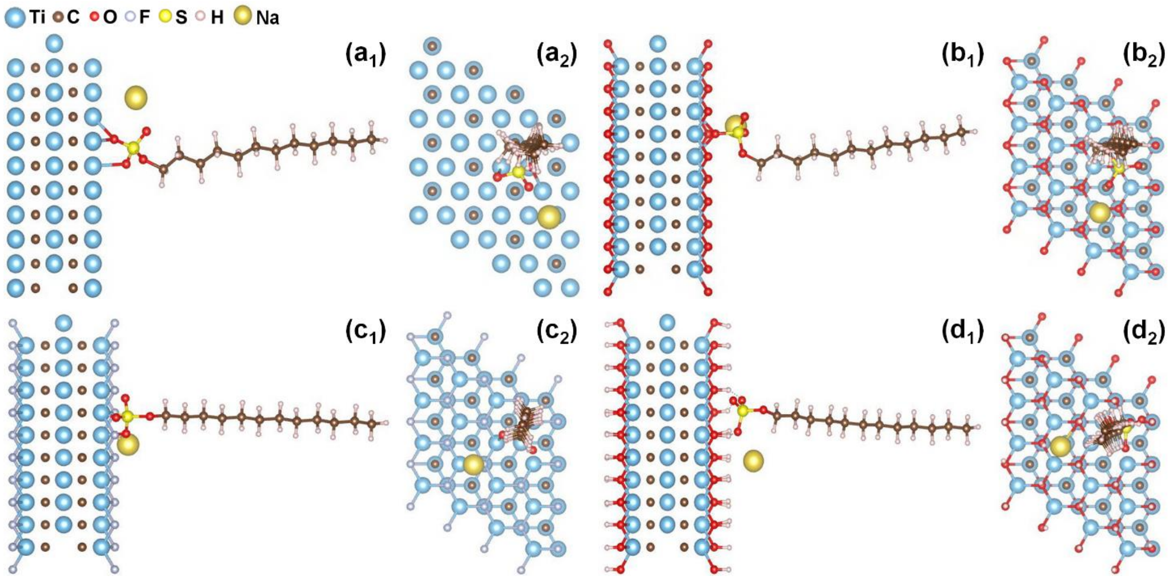

Figure S16 Side (subscript 1) and top (subscript 2) snapshots of equilibrium adsorption conformations of SDS on group-free (a) and defective $Ti_3C_2O_2$ (b), $Ti_3C_2F_2$ (c) and $Ti_3C_2(OH)_2$ (d) surfaces.

Besides E_{ads} values discussed in the main text, adsorption height (h_{ads}) is the other key item that describes the interaction between adsorbate and substrate. Combined Figure S16 and Table S3, O1 atom on SDS bind with Ti atom on Ti_3C_2 with the h_{ads} values of 2.196/2.199 Å; while, a slight larger height (2.320 Å) is found between O3 of SDS and surface Ti atom (Figure S16a$_1$). In Figure S16b$_1$, SDS^- prefers to adsorb on the defective site of $Ti_3C_2O_2$ surface via O1 atom with the h_{ads} value of 2.234/2.255 Å. Interactions between O2 and O3 atoms on SDS and Ti atoms are found in Figure S16c$_1$ for the adsorption on defective $Ti_3C_2F_2$ surface with h_{ads} values of 2.480 and 2.186 Å, respectively. Hydrogen bonds may be formed between O1/O3 on SDS and H atom on -OH terminal of $Ti_3C_2(OH)_2$, yielding the h_{ads} values of 1.823/1.658 Å.

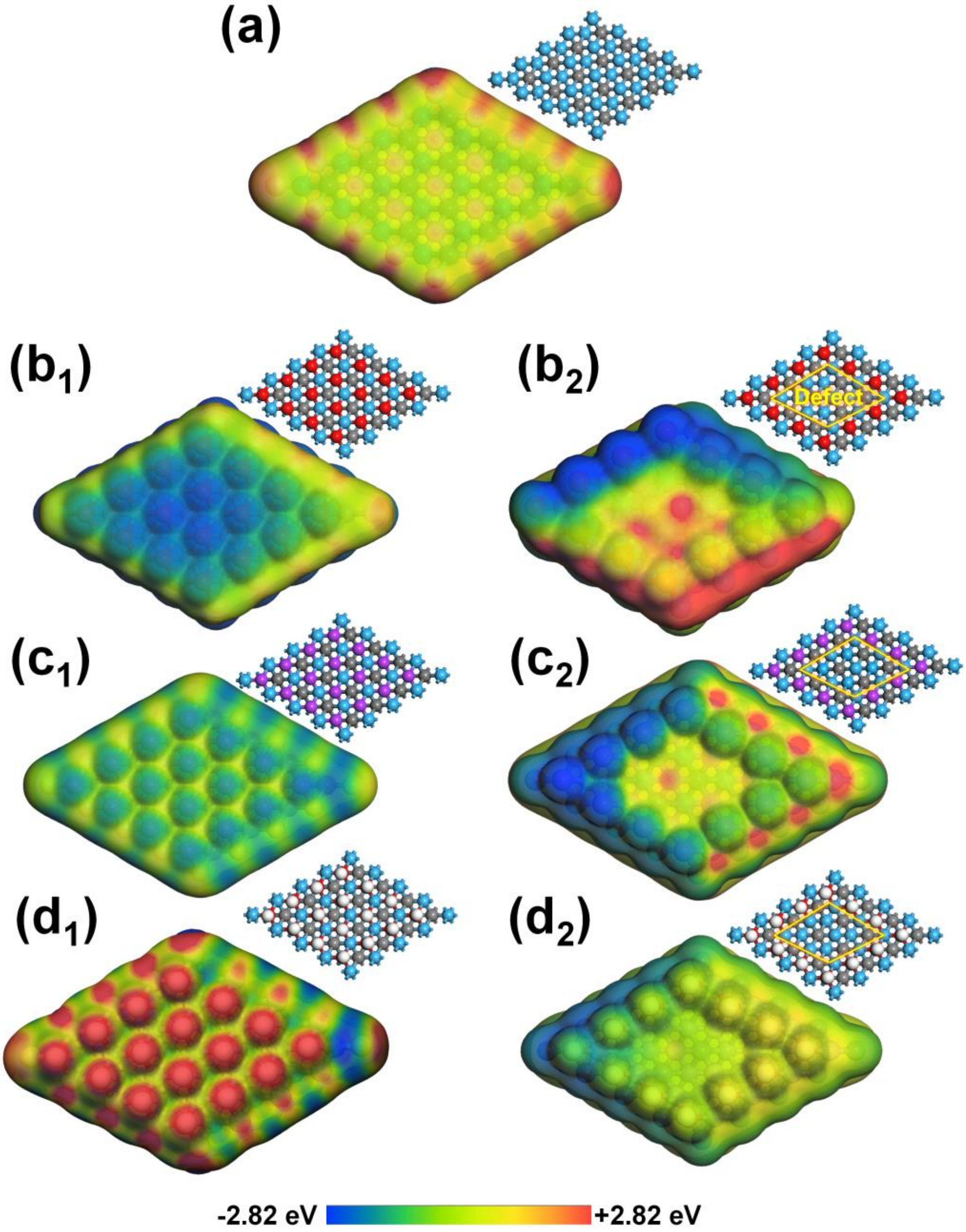

Figure S17 Electrostatic potential mapping of Ti_3C_2 (a), integrated (left) and defective (right) $Ti_3C_2O_2$ (a), $Ti_3C_2F_2$ (b) and $Ti_3C_2(OH)_2$ (c).

Table S1 Comparison of the anti-oxidative effect of sodium dodecyl sulfate with previously reported antioxidants.

No.	MXene Conc.	Antioxidant	Antioxidant Conc.	Shelf-life at RT	Ref.
1	15.0 mg/mL	Polyphosphate	100 mM (~36.8 mg/mL)	21 days	[S1]
2	0.2 mg/mL	Sodium L-ascorbate	10 mM (~2.0 mg/mL)	80 days	[S2]
		Sodium citrate	10 mM (~2.6 mg/mL)	80 days	
3	0.3 mg/mL	Sodium citrate	1.5 mg/mL	150 days	[S3]
4	2.5 mg/mL	Polyphosphate	2.5 mg/mL	15 days	[S4]
5	5 mg/mL	Sodium L-ascorbate	~60 mM (~11.9 mg/mL)	Not mentioned	[S5]
**6**	**0.05 mg/mL**	**Sodium dodecyl sulfate**	**1.5 mg/mL**	**213 days**	**This work**

At low MXene aqueous concentration, more amount of antioxidant is needed for stabilization of MXene aqueous solution.

Table S2 Semi-quantitative analysis of XPS spectra (Figure 2f) for $Ti_3C_2T_x$ after 35 days of aging without and with different concentrations of SDS.

Sample	Fraction (%)				
	Ti^{+}	Ti^{2+}	Ti^{3+}	C-Ti-F_x	TiO_2
Fresh	33.1	47.0	15.2	4.1	0.6
Fully oxidized	/	/	6.4	/	93.6
MX-0.5SDS	4.4	5.7	7.9	0.9	81.1
MX-1SDS	7.5	20.8	16.4	2.3	53.0
MX-1.5SDS	15.1	38.7	32.1	9.2	4.4
MX-2SDS	12.3	3.3	0.2	/	84.2

Table S3 Adsorption energies (E_{ads}) and distances (h_{ads}) between typical atoms for SDS on group-free and defective MXenes (Oxygen numbers are labeled in Figure S14a).

Structure	E_{ads} (eV)	Interaction atoms	h_{ads} (Å)
Ti_3C_2	-4.61	O1-Ti	2.196/2.199
		O3- Ti	2.320
Defective $Ti_3C_2O_2$	-3.09	O1-Ti	2.234/2.255
Defective $Ti_3C_2F_2$	-3.17	O2-Ti	2.480
		O3-Ti	2.186
Defective $Ti_3C_2(OH)_2$	-1.72	O1-H	1.823
		O3-H	1.658

References

[S1] V. Natu, J. L. Hart, M. Sokol, H. Chiang, M. L. Taheri, M. W. Barsoum. *Angew. Chem. Int. Edit.*, **2019**, *58*, 12655-12660.

[S2] C. W. Wu, B. Unnikrishnan, I. W. P. Chen, S. G. Harroun, H. T. Chang, C. C. Huang. *Energy Storage Mater.*, **2020**, *25*, 563-571.

[S3] X. F. Zhao, A. Vashisth, J. W. Blivin, Z. Y. Tan, D. E. Holta, V. Kotasthane, S. A. Shah, T. Habib, S. H. Liu, J. L. Lutkenhaus, M. Radovic, M. J. Green. *Adv. Mater. Interfaces*, **2020**, *7*, 2000845.

[S4] S. Huang, V. Natu, J. Tao, Y. Xia, V. N. Mochalin, M. W. Barsoum. *J. Mater. Chem. A*, **2022**, https://doi.org/10.1039/D2TA04009C.

[S5] S. Huang, V. N. Mochalin. *Inorg. Chem.*, **2022**, *61*, 9877-9887.
